# Molecular Epidemiology Reveals Genetic Diversity amongst Isolates of the *Cryptococcus neoformans*/*C. gattii* Species Complex in Thailand

**DOI:** 10.1371/journal.pntd.0002297

**Published:** 2013-07-04

**Authors:** Sirada Kaocharoen, Popchai Ngamskulrungroj, Carolina Firacative, Luciana Trilles, Dumrongdej Piyabongkarn, Wijit Banlunara, Natteewan Poonwan, Angkana Chaiprasert, Wieland Meyer, Ariya Chindamporn

**Affiliations:** 1 Mycology Laboratory, King Chulalongkorn Memorial Hospital, Faculty of Medicine, Chulalongkorn University, Bangkok, Thailand; 2 Molecular Mycology Research Laboratory, CIDM, Sydney Medical School - Westmead Hospital, The University of Sydney, Westmead Millennium Institute, Westmead, New South Wales, Australia; 3 Department of Microbiology, Faculty of Medicine Siriraj Hospital, Mahidol University, Bangkok, Thailand; 4 Grupo de Microbiología, Instituto Nacional de Salud, Bogotá, Colombia; 5 Laboratório de Micologia, Instituto de Pesquisa Clínica Evandro Chagas, Fundação Oswaldo Cruz, Rio de Janeiro, Brazil; 6 Department of Pathology, Faculty of Veterinary Sciences, Chulalongkorn University, Bangkok, Thailand; 7 Mycology Laboratory, National Institute of Health, Nonthaburi, Thailand; Fundação Oswaldo Cruz, Brazil, United States of America

## Abstract

To gain a more detailed picture of cryptococcosis in Thailand, a retrospective study of 498 *C. neoformans* and *C. gattii* isolates has been conducted. Among these, 386, 83 and 29 strains were from clinical, environmental and veterinary sources, respectively. A total of 485 *C. neoformans* and 13 *C. gattii* strains were studied. The majority of the strains (68.9%) were isolated from males (mean age of 37.97 years), 88.5% of *C. neoformans* and only 37.5% of *C. gattii* strains were from HIV patients. *URA5*-RFLP and/or M13 PCR-fingerprinting analysis revealed that the majority of the isolates were *C. neoformans* molecular type VNI regardless of their sources (94.8%; 94.6% of the clinical, 98.8% of the environmental and 86.2% of the veterinary isolates). In addition, the molecular types VNII (2.4%; 66.7% of the clinical and 33.3% of the veterinary isolates), VNIV (0.2%; 100% environmental isolate), VGI (0.2%; 100% clinical isolate) and VGII (2.4%; 100% clinical isolates) were found less frequently. Multilocus Sequence Type (MLST) analysis using the ISHAM consensus MLST scheme for the *C. neoformans/C. gattii* species complex identified a total of 20 sequence types (ST) in Thailand combining current and previous data. The Thai isolates are an integrated part of the global cryptococcal population genetic structure, with ST30 for *C. gattii* and ST82, ST83, ST137, ST141, ST172 and ST173 for *C. neoformans* being unique to Thailand. Most of the *C. gattii* isolates were ST7 = VGIIb, which is identical to the less virulent minor Vancouver island outbreak genotype, indicating Thailand as a stepping stone in the global spread of this outbreak strain. The current study revealed a greater genetic diversity and a wider range of major molecular types being present amongst Thai cryptococcal isolates than previously reported.

## Author Summary

The most common fungal pathogen of the central nervous system, members of the *Cryptococcus neoformans*/*C. gattii* species complex, causes life-threatening infection in humans with either normal or compromised immunity worldwide. The molecular epidemiology of this fungus in developing countries is highly under-investigated. Thus, three standard molecular typing methods were performed on 498 cryptococcal strains isolated from humans, the environment and animals in Thailand. As in developed countries, *C. neoformans* was found to be correlated with HIV infected patients and *C. gattii* infected humans with apparent normal immune status. Combining current and previous data, a total of 20 sequence types (ST) were identified suggesting a greater spectrum of major molecular types and genotypes of the *C. neoformans*/*C. gattii* species complex in Thailand than previously reported. Moreover, the discovery of the minor genotype (ST7) of the Vancouver Island outbreak in Thailand indicates that Asia may be an important stepping stone in its global expansion. This study gives an insight in the molecular epidemiological picture of this important fungal pathogen and necessitates further detailed studies.

## Introduction

The members of the *Cryptococcus neoformans*/*C. gattii* species complex are the causative agent of cryptococcosis, which is a systemic mycosis, in a wide range of animals and humans [1]–[4]. Inhalation of infectious propagules (basidiospores or blastoconidia) are proposed to be the source of the infection [1]. *C. neoformans* comprises two varieties and three serotypes: *C. neoformans* var. *grubii* (serotype A), *C. neoformans* var. *neoformans* (serotype D) and a hybrid (serotype AD), whereas *C. gattii* comprises two serotypes, B and C [1], [3].

Extensive surveys of the yeast have shown that the ecological niches of both species are different. *C. neoformans* has been associated worldwide with soil enriched with pigeon excreta and decaying wood [1], [5], [6]. On the contrary, *C. gattii* was until recently thought to be geographically restricted to tropical and subtropical regions and thought to be related mainly to eucalyptus trees [7]–[9]. Further environmental studies in South America and Asia pointed out several species of tropical trees as the natural habitat of *C. gattii* such as oiti (*Licania tomentosa*), almond trees (*Terminalia cattapa*), cassia (*Cassia grandis*), pottery tree (*Ficus microcarpa*) and *Syzygium cumini* [10]–[12]. A recent ongoing outbreak of *C. gattii* on Vancouver Island, Canada, a temperate area, indicated an environmental shift of this species [13]. Moreover, in contrast to previous known environmental sources of this species, *C. gattii* has been found in association with a number of native tree species (Douglas fir, alder, maple and Garry oak) on Vancouver Island rather than with eucalypt trees [13].

A number of molecular typing techniques have been used to study the molecular epidemiology of the *C. neoformans/C. gattii* species complex [14]–[17], providing more discriminatory power than conventional techniques [1]. Using M13-PCR fingerprinting, eight major molecular types have been established [18], [19]. The major molecular types VNI/AFLP1 and VNII/AFLP1A correspond to *C. neoformans* var. *grubii*, serotype A; VNIII/AFLP2 corresponds to the AD hybrid; serotype AD, and VNIV/AFLP3 corresponds to *C. neoformans* var. *neoformans*, serotype D. The molecular types VGI/AFLP4, VGII/AFLP6, VGIII/AFLP5 and VGIV/AFLP7 correspond all to *C. gattii*, serotypes B and C. These eight major molecular types have been subsequently confirmed using restriction fragment length polymorphism (RFLP) analysis of the orotidine monophosphate pyrophosphorylase (*URA5*) or phospholipase B (*PLB1*) genes, Amplified Fragment Length Polymorphism (AFLP) and Multi-Locus Sequence Typing (MLST) analysis [16], [19]–[23].

Cryptococcosis is among the most prevalent life-threatening mycoses, especially in hosts with an impaired immune system such as HIV positive patients. *C. neoformans* has been known to be the major cause of the infection in immunocompromised hosts, while the immunocompetent hosts were virtually always affected by *C. gattii* [1]. Previous studies showed that VNI is the major cause of cryptococcosis in AIDS patients, whereas VGI has been the most prevalent genotype of infections due to *C. gattii* [19]. However, in temperate climates, especially in the American continent, VGII is found to be on the rise [13], [24].

Before the AIDS era, cryptococcosis in Thailand was mainly caused by *C. gattii*, serotypes B and C [25]. At that time, the serotypes A and D were reported as opportunistic fungal pathogens, which were mainly related to pet bird excreta [26]. *C. gattii* has never been reported from the environment in Thailand [25]–[27]. The prevalence of cryptococcosis has increased dramatically since 1992 due to a rising number of AIDS patients, with *C. neoformans* var. *grubii* serotype A being the major cause of infection [27]–[31]. Two typing methods, random amplification of polymorphic DNA (RAPD) [27], [30], [32] and Pulsed Field Gel Electrophoresis (PFGE) [32], were previously applied for the genotyping of Thai isolates, which revealed the prevalence of serotype A strains. A recent molecular epidemiological study of 183 Thai isolates using MLST analysis showed that almost all of the isolates belonged to the VNI molecular type, with only one isolate being found to be VNII [33]. However, the genetic diversity of Thai isolates is expected to be more diverse since *C. neoformans* var. *neoformans* (VNIV) and *C. gattii* (VG) strains had been isolated before in Thailand [26], [30]. Thus, the current study aimed to expend the current epidemiological knowledge by including a further 498 clinical, environmental and veterinary isolates of the *C. neoformans/C. gattii* species complex.

## Methods

### Strains and media

The 498 cryptococcal isolates were recovered from the culture collections of the Molecular Mycology and Mycobacteriology Laboratory, Faculty of Medicine, Siriraj Hospital, Mahidol University, the Mycology Unit Laboratory, King Chulalongkorn Memorial Hospital, Faculty of Medicine, Chulalongkorn University, the Mycology Laboratory of the National Institute of Health, Nonthaburi, Thailand [30] and the Molecular Mycology Research Laboratory, Westmead Hospital, Westmead, NSW, Australia (see Table S1). All strains were maintained in glycerol at −80°C.

### Environmental sampling

The environmental strains were collected from pigeon droppings in Bangkok during the years 2002–2005 using a method described previously [26]. Briefly, the pigeon droppings were collected into sterile zip-lock bags from 21 districts in Bangkok and transferred to the Mycology Unit Laboratory, King Chulalongkorn Memorial Hospital for isolation and identification. The pigeon droppings were dissolved in 0.85% normal saline, vigorously vortex and centrifuged. The supernatant was diluted, then spread onto Sabouraud Dextrose Agar plates supplemented with 40 µg/mL chloramphenicol and incubated at 37°C and observed for growth of yeast colonies every day. The grown yeast colonies were transferred to new media and identified as *C. neoformans* via india-ink preparation, urease test and phenoloxidase production test [34]. If the isolates were positive for all three tests they were preliminarily identified as belonging to the *C. neoformans/C. gattii* species complex and included into the study. Two additional environmental strains isolated from pigeon droppings were kindly provided from the mycology laboratory, Chiang Mai University. The strain information is listed in Table S1.

### *URA5*-RFLP

Genomic DNA was isolated as described previously [19]. The major molecular types were determined by *URA5*-RFLP analysis as previously described [19]. Briefly, the *URA5* gene was amplified with the following primers URA5 (5′ATGTCCTCCCAAGCCCTCGACTCCG3′) and SJ01 (5′TTAAGACC TCTGAACACCGTACTC3′). The obtained amplification products were digested with the restriction enzymes *Hha*I and *Sau96*I. The digested PCR products were visualized and compared to the reference strains on a 3% agarose gel after staining with ethidium bromide.

### PCR-fingerprinting

PCR-fingerprinting was carried out as described previously [18] using the microsatellite specific primer M13 (5′ GAGGGTGGCGGTTCT 3′). The PCR-fingerprinting profiles were visualized and compared on 1.4% agarose gels containing ethidium bromide using the 1D gel analysis module BioGalaxy in the software package BioloMICS ver. 7.5.30 (BioAware, Hannut, Belgium). Strains with identical M13 PCR-fingerprints were grouped in the same M13 type (see Table S1).

### MLST

VNI strains representative of the different M13 types identified by PCR-fingerprinting and all VNII, VNIV and VGII strains were chosen for MLST analysis. Using the ISHAM consensus MLST typing scheme, seven unlinked genetic loci, including conserved and variable regions of *CAP59*, *GPD1*, *LAC1*, *PLB1*, *SOD1*, *URA5* and the IGS1 region, were amplified using the primers and amplification parameters described by the ISHAM Cryptococcal Working Group [21], sequenced and analyzed as reported previously [35], [36]. To put the newly identified molecular patterns into context with previous Thai studies, sequences of additional strains of the *C. neoformans/C. gattii* species complex were retrieved from those studies [33], [37] (see Tables S2 and S3). The previously published Thai sequence types [33] were downloaded from the mlst.mycologylab.org webpage. The generated sequences were manually edited using the software Sequencher 4.9 (Gene Codes Corporation, MI, USA) and aligned using Clustal W [38], part of the program Bioedit 7.0.9.0 [39]. The concatenated alignments were then imported to the program MEGA 5.03 [40] and analyzed using the neighbor-joining method with p-distance [41]. Bootstrap analysis [42] using 1000 replicates was used to estimate support for clades of the concatenate dataset. The genetic network analysis was performed using the software Network 4.5.1.6 (Fluxus Technologies Ltd., Suffolk, UK). All allele types and subsequently the combined sequence types were assigned using the ISHAM consensus database at mlst.mycologylab.org, as described previously [35]. All MLST sequences are deposited at mlst.mycologylab.org. The sequences of the herein determined alleles of the seven MLST loci are deposited in GenBank (Table S4).

### Reference strains

The following set of laboratory standard reference strains representing each of the eight major molecular type of the *Cryptococcus neoformans/C. gattii* species complex were used: WM 148 (serotype A, VNI/AFLP1), WM 626 (serotype A, VNII/AFLP1A), WM 628 (serotype AD, VNIII/AFLP2), WM629 (serotype D, VNIV/AFLP3), WM 179 (serotype B, VGI/AFLP4), WM 178 (serotype B, VGII/AFLP6), WM 175 (serotype B, VGIII/AFLP5) WM 779 (serotype C, VGIV/AFLP7), R265 (VGIIa) and R272 (VGIIb) [19], [35].

### Patient data

Demographic and clinical data for each isolate, including isolation site and date, patient's residence, age, gender and HIV status, were retrieved from the clinical records if they were available and are listed in Table S1. These isolates are anonymous and the data cannot be used to trace back to individuals

### Data analysis

Statistical analysis was performed using the SPSS software package ver. 18.0.0 (IBM, Armonk, New York). Unknown data were regarded as missing data and excluded from the calculations.

## Results

### Demographic data

Among the 498 strains collected, 386, 83 and 29 strains were from clinical, environmental and veterinary sources, respectively. Of the clinical strains, 68.9% were from male and 31.1% from female patients, with an average age of 37.97 years. A total of 485 *C. neoformans* and 13 *C. gattii* strains were studied. Most of the *C. neoformans* clinical strains were from HIV positive patients (88.5%). In comparison, only 37.5% of the *C. gattii* strains were from HIV positive patients. The clinical strains were collected from all areas of Thailand with most strains originating from Bangkok (47.3%) and the central part of Thailand (27.0%). From the clinical strains, 80% were recovered from CSF, 17.5% from blood and 2.5% from other sites. All environmental isolates were obtained from pigeon droppings. The most common site of cryptococcal isolation in veterinary cases was the nasal cavity of cats (72.4%). For further information, see Table S1.

### Major molecular types

To examine the genetic diversity of Thai cryptococcal strains, the major molecular types were determined by M13 PCR-fingerprinting and/or *URA5*-RFLP analysis [19]. As seen globally [43], VNI was the most common molecular type among Thai human (94.6%), environmental (98.8%) and animal (86.2%) isolates, though less frequent, VNII, VNIV, VGI and VGII were also found (Table 1).

**Table 1:** Distribution of the major molecular types among strains of*Cryptococcus neoformans* and *C. gattii* from different sources in Thailand.

Source	Molecular type					Total
	VNI	VNII	VNIV	VGI	VGII	
**Human**	365 (94.6%)	8 (2.1%)	0	1 (0.3%)	12 (3.1%)	386
**Environment**	82 (98.8%)	0	1 (1.2%)	0	0	83
**Animal**	25 (86.2%)	4 (13.8%)	0	0	0	29
**Total**	**472 (94.8%)**	**12 (2.4%)**	**1 (0.2%)**	**1 (0.2%)**	**12 (2.4%)**	**498**

### M13 PCR-fingerprinting of VNI Thai isolates showed six different subtypes

As the majority of VNI Thai isolates were reported previously to be clonal [33], M13 PCR fingerprinting analysis, which has shown to differentiate cryptococcal molecular subtypes in several previous studies [13], [18], [37], was performed to check for clonality amongst the collected Thai VNI isolates. The obtained PCR fingerprinting patterns were assigned with a M13 type. Six M13 types (A, B, C, D, E, and F) were identified amongst all studied isolates, with type A being the most common type identified (90.3%) (Table 2). The genetic diversity identified by M13 PCR-fingerprinting analysis suggested that the Thai isolates are more genetically diversified than previously reported [33].

**Table 2:** Distribution of the M13 types among Thai VNI isolates.

M13 type	Frequency
A	426 (90.3%)
B	1 (0.2%)
C	3 (0.6%)
D	9 (1.9%)
E	1 (0.2)
F	32 (6.8%)
**Total**	**472**

### Thai*C. neoformans* isolates are genetically diversified

**Figure 1:**
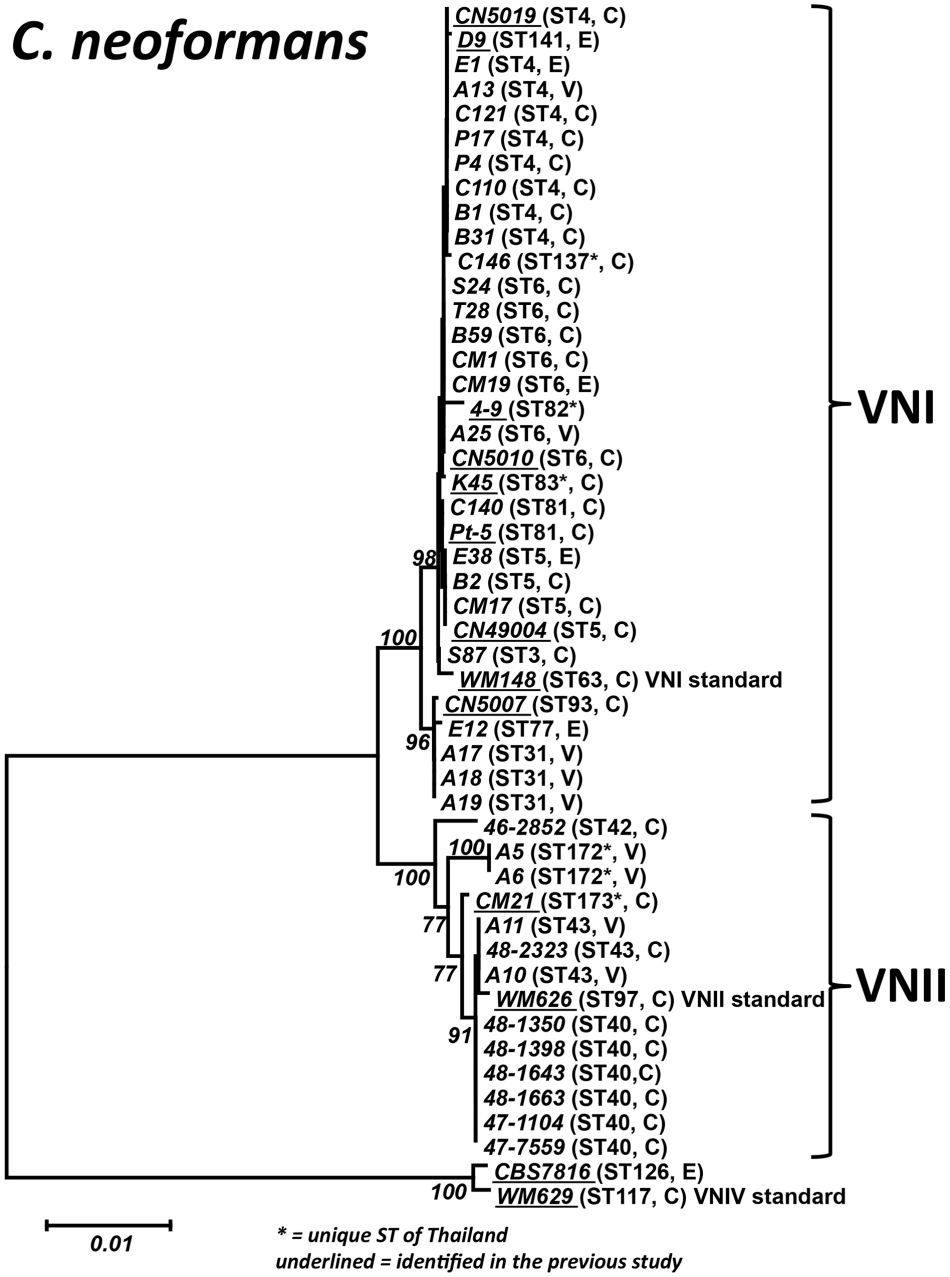
Phylogram of the Thai*C.* *neoformans* isolates. Phylogram depicting the genetic relationships between the Thai *C. neoformans* isolates based on neighbor joining analysis of the concatenated seven ISHAM consensus MLST loci using the program MEGA 5.03. Bold numbers on the branches indicate bootstrap support above 75%. Underlined strain numbers indicate STs identified in a previous study [33]. C = clinical, E = environmental, V = veterinary.

**Figure 2:**
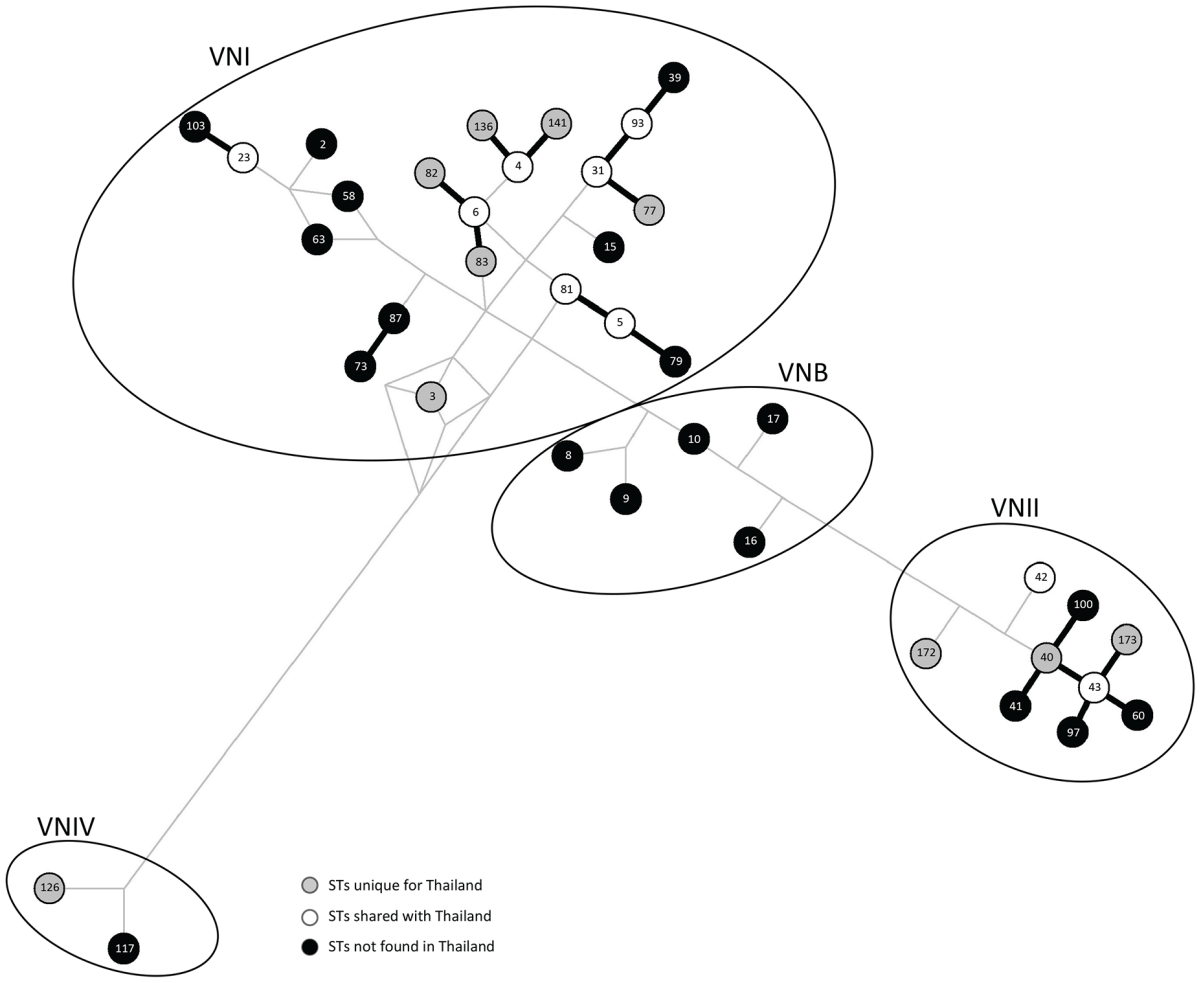
Gene network placing the Thai*C.* *neoformans* isolates in global context. Gene network constructed from all *C. neoformans* ST types identified by MLST analysis in the current study in-cooperating the STs previously obtained from Thai cryptococcal isolates [33] and standard strains based on the combined seven ISHAM consensus MLST loci using the program Network 4.5.1.6, showing the close relationships between STs present in Thailand and globally.

To verify the obtained diversity and to enable comparison with previous studies, MLST analysis, which has a superior discriminatory power and reproducibility over M13 PCR-fingerprinting [37], was performed. MLST analysis was performed on representative strains of each M13 type from the VNI isolates (14 strains of M13 type A, 1 of M13 type B, 2 of M13 type C, 2 of M13 type D, 1 of M13 type E, and 4 of M13 F) and for all VNII and VNIV isolates. Eight additional sequence types (STs), with ST3, ST31, ST77, and ST137 for VNI and ST40, ST42, ST43 and ST172 for VNII (Table 3, Table S1 and Figure 1), were identified amongst the studied *C. neoformans* isolates when compared with a previous report, which had identified the following STs: ST4, ST5, ST6, ST81, ST82, ST83, ST85, ST93 and ST141 for VNI; and ST173 for VNII [33]. Network analysis showed that the Thai *C. neoformans* isolates are an integral part of the global population structure of this species, with nine ST's being unique to Thailand, but closely related to other global isolates (Figure 2).

**Table 3:** Allele types and sequence types of selected Thai cryptococcal isolates.

Strain name	Mol. type	Source	M13 type	*CAP59*	*GPD1*	*IGS1*	*LAC1*	*PLB1*	*SOD1*	*URA5*	ST
47-2158	VGII	Human	ND	2	6	10	4	2	15	2	**7**
47-4995	VGII	Human	ND	2	6	10	4	2	15	2	**7**
47-5055	VGII	Human	ND	2	6	10	4	2	15	2	**7**
47-5061	VGII	Human	ND	2	6	10	4	2	15	2	**7**
DMST20763	VGII	Human	ND	2	6	10	4	2	15	2	**7**
DMST20764	VGII	Human	ND	2	6	10	4	2	15	2	**7**
DMST20765	VGII	Human	ND	2	6	10	4	2	15	2	**7**
DMST20766	VGII	Human	ND	2	6	10	4	2	15	2	**7**
DMST20767	VGII	Human	ND	2	6	10	4	2	15	2	**7**
DMST20768	VGII	Human	ND	2	6	10	4	2	15	2	**7**
MC-S-265	VGII	Human	ND	2	6	10	4	2	15	2	**7**
MC-S-115	VGII	Human	ND	2	6	32	4	2	15	2	**30**
A13	VNI	Animal	A	1	1	1	4	2	1	5	4
B1	VNI	Human	A	1	1	1	4	2	1	5	4
C110	VNI	Human	A	1	1	1	4	2	1	5	4
C121	VNI	Human	A	1	1	1	4	2	1	5	4
E1	VNI	Environment	A	1	1	1	4	2	1	5	4
P17	VNI	Human	A	1	1	1	4	2	1	5	4
P4	VNI	Human	A	1	1	1	4	2	1	5	4
A25	VNI	Animal	A	1	1	1	3	2	1	5	6
B59	VNI	Human	A	1	1	1	3	2	1	5	6
CM1	VNI	Human	A	1	1	1	3	2	1	5	6
CM19	VNI	Environment	A	1	1	1	3	2	1	5	6
S24	VNI	Human	A	1	1	1	3	2	1	5	6
T28	VNI	Environment	A	1	1	1	3	2	1	5	6
C146	VNI	Human	A	1	3	1	4	2	1	5	**137**
A19	VNI	Animal	B	1	1	10	3	2	1	1	**31**
A17	VNI	Animal	C	1	1	10	3	2	1	1	**31**
A18	VNI	Animal	C	1	1	10	3	2	1	1	**31**
S87	VNI	Human	D	1	1	1	3	4	1	1	**3**
E12	VNI	Environment	D	1	1	25	3	2	1	1	**77**
B31	VNI	Human	E	1	1	1	4	2	1	5	4
B2	VNI	Human	F	1	3	1	5	2	1	1	5
CM17	VNI	Human	F	1	3	1	5	2	1	1	5
E38	VNI	Environment	F	1	3	1	5	2	1	1	5
C140	VNI	Human	F	1	1	1	5	2	1	1	81
47-1104	VNII	Human	ND	2	9	14	8	11	12	4	**40**
47-7559	VNII	Human	ND	2	9	14	8	11	12	4	**40**
48-1350	VNII	Human	ND	2	9	14	8	11	12	4	**40**
48-1398	VNII	Human	ND	2	9	14	8	11	12	4	**40**
48-1643	VNII	Human	ND	2	9	14	8	11	12	4	**40**
48-1663	VNII	Human	ND	2	9	14	8	11	12	4	**40**
46-2852	VNII	Human	ND	8	10	15	8	12	3	11	**42**
48-2323	VNII	Human	ND	2	9	14	8	11	11	4	**43**
A10	VNII	Animal	ND	2	9	14	8	11	11	4	**43**
A11	VNII	Animal	ND	2	9	14	8	11	11	4	**43**
A5	VNII	Animal	ND	2	9	14	11	11	16	15	172
A6	VNII	Animal	ND	2	9	14	11	11	16	15	172
CBS7816	VNIV	Environment	ND	17	21	28	19	14	1	20	**126**

ND = not done, ST = sequence type, Mol. = molecular, bold = new ST types of Thai isolates identified in this study.

### Almost all clinical VGII isolates belong to the same ST as the low virulent Vancouver Island outbreak strain

**Figure 3:**
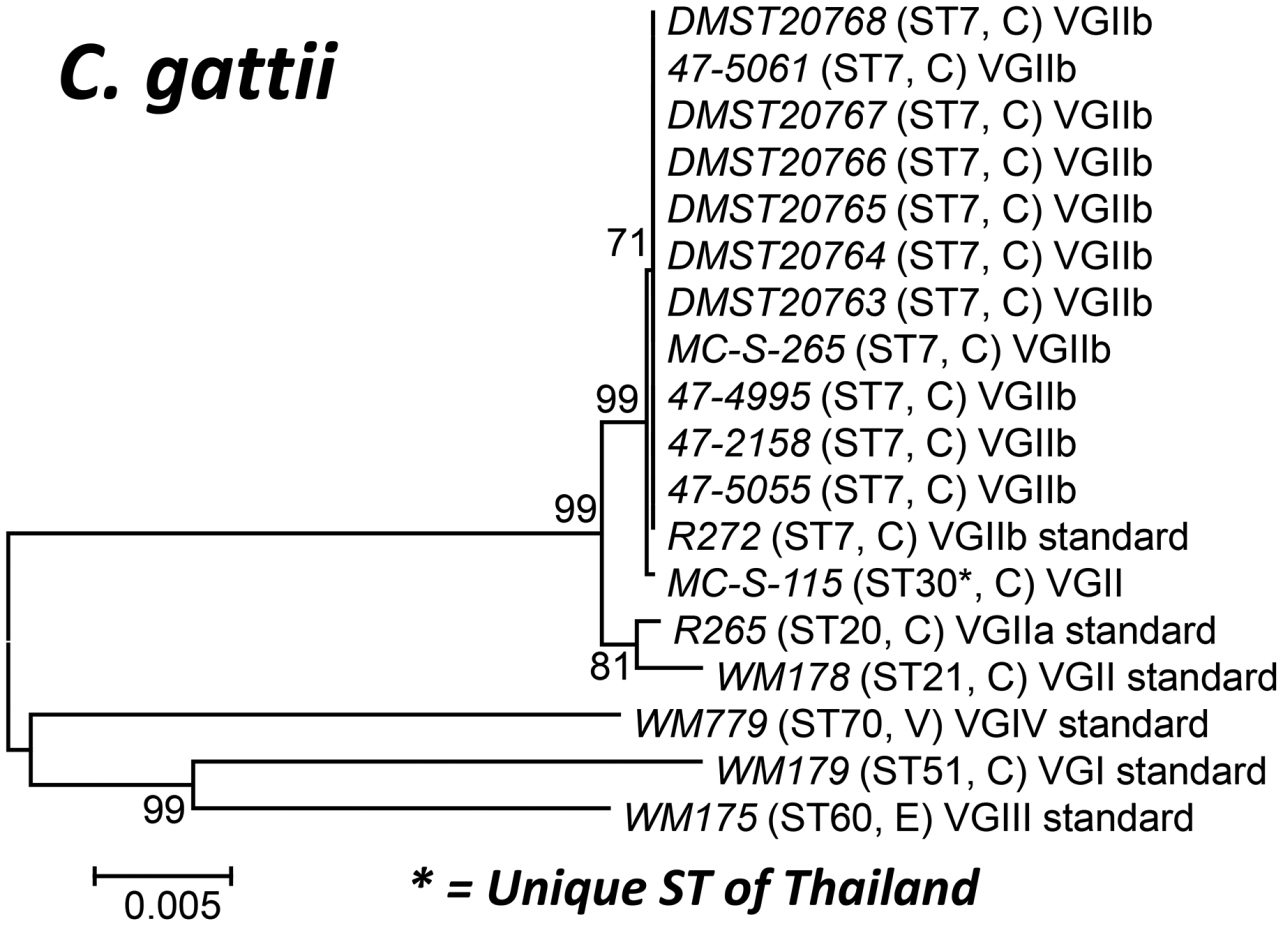
Phylogram of the Thai*C.* *gattii* isolates. Phylogram depicting the genetic relationships between the Thai *C. gattii* isolates based on neighbor joining analysis of the concatenated seven ISHAM consensus MLST loci using the program MEGA 5.03. Bold numbers on the branches indicate bootstrap support above 75%. C = clinical, E = environmental.

As VGII isolates are the causative agent of the ongoing outbreaks on Vancouver Island, Canada and Pacific Northwest region of USA [35], [44], MLST analysis was performed to determine the relationship of the Thai VGII isolates to the outbreak strains. Surprisingly, 11 out of the 12 VGII isolates were identical to the genotype of the low virulent Vancouver Island outbreak strains, VGIIb/ST7 (Figure 3). One isolate had a ST, which was unique to Thailand (ST30) (see Tables 3 and S1 and Figure 3).

## Discussion

The obtained data concerning the demographics and the HIV status of the patients were in line with previous reports of cryptococcosis from Thailand [25], [31]. Besides some missing demographic data it is clear from the available data that *C. neoformans* was the most common species identified among HIV positive patients, while *C. gattii* was mainly a primary pathogen in immunocompetent patients, which is in accordance with a previous global study [18]. The fact that most isolates were recovered from male HIV positive patients with an average age of infection of 37.97 years represents the HIV demography in Thailand, with 60% of the HIV infected patients being male with an average age of 30–34 years [45].

*C. neoformans* has been found worldwide, with VNI being the most common molecular type, including recent reports from Thailand [33]. The molecular typing in the current study confirmed this paradigm, where VNI is predominant regardless of the isolate source. Moreover, the rare molecular type VNII, for which only one isolate had been reported previously from Thailand [33], has now been identified from several strains from both humans and animals. As the natural reservoir of VNII has never been reported, the herein presented data allow to suggest: that a close relationship between animal and human VNII isolates may exist, as strains from humans and animals share the same genotype, ST43 (Figure 1 and Table 3). However, further studies are needed to draw a definite conclusion as the numbers of the studied VNII strains are very small and other human VNII strains showed no relationships with animal strains. On the other hand, a strong relationship between VNI clinical and environmental strains is evident, as they share the same STs (Figure 1 and Table 3).

The correlation between *C. neoformans* and HIV in Thailand is supported by the low prevalence of the genotype VNIc/M5, corresponding to M13 type F in the current study, which is known to be associated with non-HIV patients in China [46], Korea [37] and Japan [36]. In fact, only 6% (23 out of 386 isolates) of the clinical cases were of M13 type F (VNIc/M5) (Figure S1) and only one of them (P21) had been isolated from a HIV positive patient. All other cryptococcal isolates form HIV positive patients had either the M13 type A or D (Table S1).

The herein obtained MLST data when combined with data previously reported [33] showed clearly, that the STs present in Thailand are an integral part of the global population genetic structure, and are not as unique as previously reported [33]. For the *C. neoformans* molecular type VNI, seven of the STs are shared with global strains and six are unique for Thailand (Figure 2). All of the isolates form a close network with a number of Thai specific STs and are directly linked to other globally present STs (Figure 2).

The current study describes for the first time molecular typing of *C. gattii* isolates from Thailand, taken into account the literature since the 1990's [30], [31]. The high percentage of the VGII molecular type (92.3%) amongst the studied *C. gattii* isolates is in contrast to a report from a neighboring country, Malaysia, where 76.5% of the *C. gattii* isolates belonged to the molecular type VGI [47]. No VGIII or VGIV isolates have been found in the current study and the fact that they have never been reported from this region may suggest that those molecular types are not endemic in this area. The geographically closest related place from which VGIII and VGIV isolates have been reported is India [43], [48].

Before the AIDS epidemic, a predominance of *C. gattii* as the causative agent of cryptococcosis was found in Thailand, which was possibly related to non-HIV immunocompromised conditions [25], [26]. A recent study on non-HIV cryptococcosis cases suggested that the disease was not such a rare event in HIV negative patients and is also associated with high mortality rates [49], [50], a fact also seen with the cases of *C. gattii* infection investigated in the current study.

Moreover, the predominance of the VGII molecular type in this tropical region revealed in the current study is of special interest, as a similar situation was only described form the northern part of Brazil [51], which is in contrast to most of the described isolations which are associated mainly with areas of temperate climate or in the high mountain regions of Colombia [24], [52], [53]. In addition, the fact that several strains (the DMST strains) were isolated more than 10 years ago (Table S1), [30], [54] suggests that this molecular type is prevalent in Thailand, as it is in South America [19], but unlike Australia [55] or Europe [56] where the molecular type VGI is predominant.

The fact that 11 out of the 12 *C. gattii* strains studied showed an identical ST type to the one of the Vancouver Island outbreak strain, VGIIb = ST7 is remarkable. It reveals the high clonality that this VGIIb *C. gattii* population has in Thailand, which is similar to the situation described in Australia [35]. It confirms the point previously made, that this low virulent outbreak strain is globally present, with Australia and Thailand being important stepping-stones in the global spread of this outbreak strain, linking South America, via Australia with North America and Europe.

In summary, as in other worldwide studies, the same distribution of cryptococcal genotypes has been found in Thailand, with a predominance of *C. neoformans* var. *grubii*, molecular type VNI, isolated from HIV positive patients. Our study suggests a greater genetic diversity among Thai cryptococcal isolates especially amongst VNI strains with 13 different STs than reported previously [33]. The majority of Thai *C. gattii* isolates are clonal and identical to the Vancouver Island outbreak strain with VGIIb = ST7, identifying Thailand as a stepping-stone in its global spread. In addition, a strong linkage between environmental and clinical strains was found for the VNI isolates. A connection between other rare molecular types, such as VNII for *C. neoformans* or VGI and VGII for *C. gattii* and the environment in Thailand could still not be found and needs further investigation. Extensive environmental and veterinary sampling would be of great help to fill this gap. Moreover, despite an advanced development of HIV treatment, cryptococcosis is still a major problem as an opportunistic infection in Thailand, making further studies, concerning the epidemiology and virulence of the *Cryptococcus neoformans/C. gattii* species complex mandatory for a proper management of the disease in the future.

## Supporting Information

Figure S1 **Phylogram correlating the newly identified Thai** ***C. neoformans*** **sequence types with previously reported types.** Phylogram depicting the genetic relationships between the Thai VNI isolates studied herein in combination with previously published data representing the following M13 PCR-fingerprinting patterns VNIa, VNIb, VNIc/M5 and VNId (32) based on neighbor joining analysis of the concatenated seven ISHAM consensus MLST loci. Bold numbers on the branches indicate bootstrap support above 75%. Letters in brackets indicate the M13 type. WM148 = VNI standard, WM626 = VNII standard.
(TIF)

Table S1 **Strains used in this study and associated demographic and molecular data.**
(DOC)

Table S2 **Correlation between old and new allele and sequence type numbering from the Simwami** ***et al.*** **2011 (28) publication and the new** ***C. gattii*** **MLST database at mlst.mycologylab.com for the MLST data used in the current study.**
(DOC)

Table S3 **MLST data for the additional published** ***C. neoformans*** **strains used in this study.**
(DOC)

Table S4 **GenBank accession numbers for all sequences of the MLST alleles obtained from Thai** ***C. neoformans*** **and** ***C. gattii*** **isolates used in this study.**
(DOC)
