# Supplementary figures and images for: Molecular Epidemiology Reveals Genetic Diversity amongst Isolates of the Cryptococcus neoformans/C. gattii Species Complex in Thailand
Source: PLoS Negl Trop Dis. 2013 Jul 4;7(7):e2297. doi: 10.1371/journal.pntd.0002297 (PMC3701708; doi:10.1371/journal.pntd.0002297)

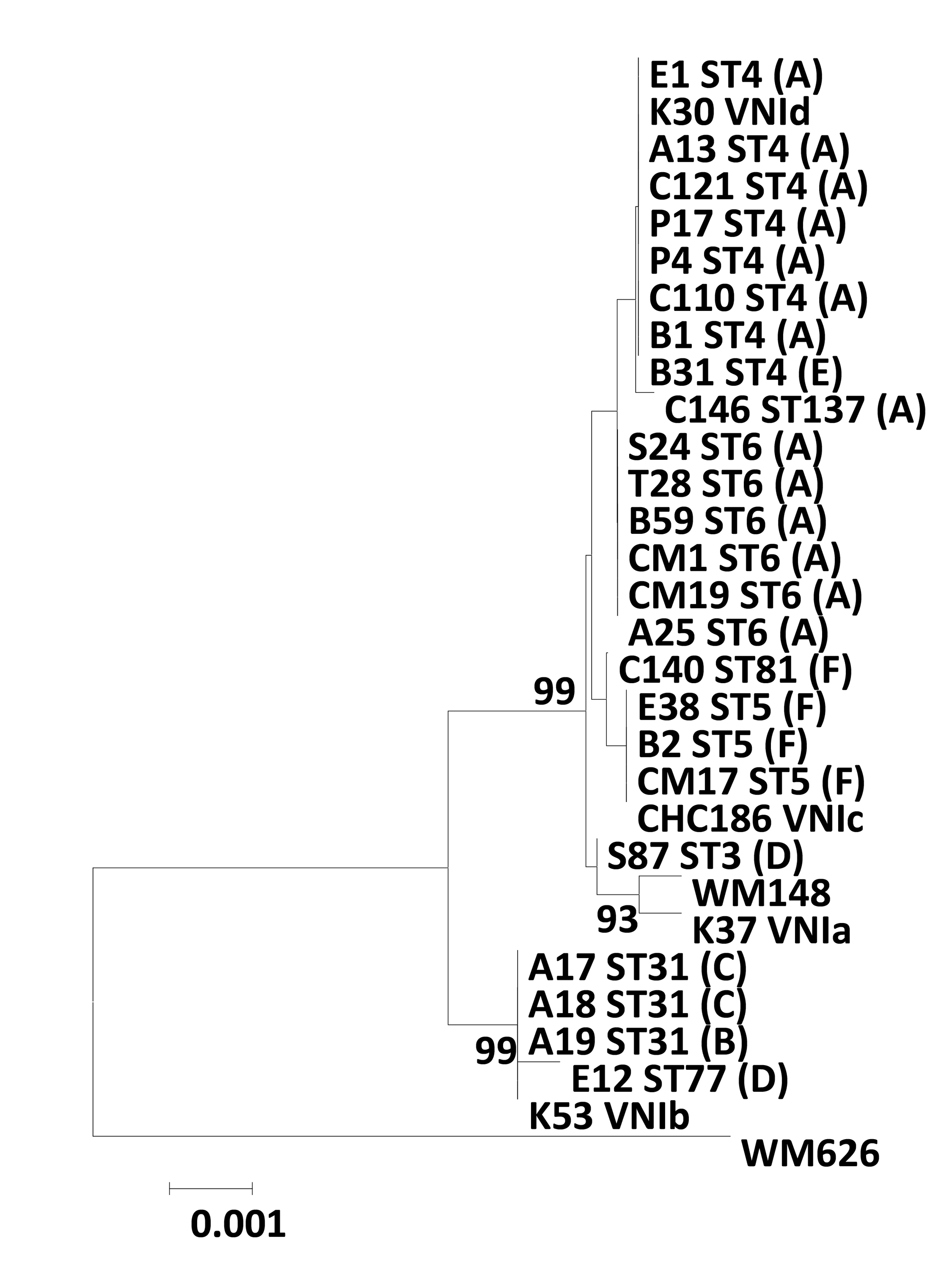

Supplement: Figure S1 — Phylogram correlating the newly identified Thai C. neoformans sequence types with previously reported types. Phylogram depicting the genetic relationships between the Thai VNI isolates studied herein in combination with previously published data representing the following M13 PCR-fingerprinting patterns VNIa, VNIb, VNIc/M5 and VNId (32) based on neighbor joining analysis of the concatenated seven ISHAM consensus MLST loci. Bold numbers on the branches indicate bootstrap support above 75%. Letters in brackets indicate the M13 type. WM148 = VNI standard, WM626 = VNII standard. (TIF) [file pntd.0002297.s001.tif]
