# Supplementary material for: Molecular Epidemiology Reveals Genetic Diversity amongst Isolates of the Cryptococcus neoformans/C. gattii Species Complex in Thailand
Source: PLoS Negl Trop Dis. 2013 Jul 4;7(7):e2297. doi: 10.1371/journal.pntd.0002297 (PMC3701708; doi:10.1371/journal.pntd.0002297)
Supplement: Table S1 — Strains used in this study and associated demographic and molecular data. (DOC) [file pntd.0002297.s002.doc]

**Table S1.** Strains used in this study and associated demographic and molecular data

| **Strain No.** | **Date of isolation** | **Source** | **Site** | **Age** | **Gender** | **HIV status** | **Province of Thailand*** | **Molecular type** | **M13 type** | **MLST type** |
| --- | --- | --- | --- | --- | --- | --- | --- | --- | --- | --- |
| 47-4932 | 2004 | Clinical | CSF |  | M |  |  | VGI |  |  |
| 47-2158 | 2004 | Clinical | CSF | 60 | M | Negative | Bangkok | VGIIb |  | ST7 |
| 47-4995 | 2004 | Clinical | CSF | 58 | F | Negative | Prachuapkeerikun | VGIIb |  | ST7 |
| 47-5055 | 2004 | Clinical | Ventriculostomy tube | 57 | F | Negative | Prachuapkeerikun | VGII |  | ST7 |
| 47-5061 | 2004 | Clinical | CSF | 58 | F | Negative | Prachuapkeerikun | VGIIb |  | ST7 |
| DMST20763 | 1994 | Clinical | CSF |  |  |  |  | VGIIb |  | ST7 |
| DMST20764 | 1994 | Clinical | CSF |  |  |  |  | VGIIb |  | ST7 |
| DMST20765 | 1994 | Clinical | CSF |  |  |  |  | VGIIb |  | ST7 |
| DMST20766 | 1994 | Clinical | CSF |  |  | Positive |  | VGIIb |  | ST7 |
| DMST20767 | 1995 | Clinical | CSF |  |  |  |  | VGIIb |  | ST7 |
| DMST20768 | 1994 | Clinical | Blood |  |  | Positive |  | VGIIb |  | ST7 |
| MC-S-115 | 1993 | Clinical | CSF |  | M | Positive |  | VGII |  | ST30* |
| MC-S-265 | 1995 | Clinical | CSF |  | M | Negative |  | VGIIb |  | ST7 |
| A13 | 2005 | Veterinary | Nose |  |  |  |  | VNI | A | ST4 |
| A14 | 2005 | Veterinary | Nose |  |  |  |  | VNI | A |  |
| A15 | 2005 | Veterinary | Nose |  |  |  |  | VNI | A |  |
| A16 | 2005 | Veterinary | Nose |  |  |  |  | VNI | A |  |
| A21 | 2005 | Veterinary | Nose |  |  |  |  | VNI | A |  |
| A22 | 2005 | Veterinary | Nose |  |  |  |  | VNI | A |  |
| A23 | 2005 | Veterinary | Nose |  |  |  |  | VNI | A |  |
| A24 | 2005 | Veterinary | Nose |  |  |  |  | VNI | A |  |
| A25 | 2005 | Veterinary | Nose |  |  |  |  | VNI | A | ST6 |
| A26 | 2005 | Veterinary | Nose |  |  |  |  | VNI | A |  |
| A27 | 2005 | Veterinary | Nose |  |  |  |  | VNI | A |  |
| A28 | 2005 | Veterinary | Nose |  |  |  |  | VNI | A |  |
| A8 | 2005 | Veterinary | Nose |  |  |  |  | VNI | A |  |
| B1 |  | Clinical |  |  |  |  |  | VNI | A | ST4 |
| B10 |  | Clinical |  |  |  |  |  | VNI | A |  |
| B11 |  | Clinical |  |  |  |  |  | VNI | A |  |
| B13 |  | Clinical |  |  |  |  |  | VNI | A |  |
| B14 |  | Clinical |  |  |  |  |  | VNI | A |  |
| B15 |  | Clinical |  |  |  |  |  | VNI | A |  |
| B16 |  | Clinical |  |  |  |  |  | VNI | A |  |
| B17 |  | Clinical |  |  |  |  |  | VNI | A |  |
| B18 |  | Clinical |  |  |  |  |  | VNI | A |  |
| B19 |  | Clinical |  |  |  |  |  | VNI | A |  |
| B20 |  | Clinical |  |  |  |  |  | VNI | A |  |
| B21 |  | Clinical |  |  |  |  |  | VNI | A |  |
| B22 |  | Clinical |  |  |  |  |  | VNI | A |  |
| B23 |  | Clinical |  |  |  |  |  | VNI | A |  |
| B25 |  | Clinical |  |  |  |  |  | VNI | A |  |
| B26 |  | Clinical |  |  |  |  |  | VNI | A |  |
| B27 |  | Clinical |  |  |  |  |  | VNI | A |  |
| B28 |  | Clinical |  |  |  |  |  | VNI | A |  |
| B29 |  | Clinical |  |  |  |  |  | VNI | A |  |
| B30 |  | Clinical |  |  |  |  |  | VNI | A |  |
| B32 |  | Clinical |  |  |  |  |  | VNI | A |  |
| B33 |  | Clinical |  |  |  |  |  | VNI | A |  |
| B34 |  | Clinical |  |  |  |  |  | VNI | A |  |
| B35 |  | Clinical |  |  |  |  |  | VNI | A |  |
| B36 |  | Clinical |  |  |  |  |  | VNI | A |  |
| B37 |  | Clinical |  |  |  |  |  | VNI | A |  |
| B38 |  | Clinical |  |  |  |  |  | VNI | A |  |
| B39 |  | Clinical |  |  |  |  |  | VNI | A |  |
| B40 |  | Clinical |  |  |  |  |  | VNI | A |  |
| B41 |  | Clinical |  |  |  |  |  | VNI | A |  |
| B43 |  | Clinical |  |  |  |  |  | VNI | A |  |
| B44 |  | Clinical |  |  |  |  |  | VNI | A |  |
| B45 |  | Clinical |  |  |  |  |  | VNI | A |  |
| B46 |  | Clinical |  |  |  |  |  | VNI | A |  |
| B47 |  | Clinical |  |  |  |  |  | VNI | A |  |
| B49 |  | Clinical |  |  |  |  |  | VNI | A |  |
| B50 |  | Clinical |  |  |  |  |  | VNI | A |  |
| B53 |  | Clinical |  |  |  |  |  | VNI | A |  |
| B55 |  | Clinical |  |  |  |  |  | VNI | A |  |
| B56 |  | Clinical |  |  |  |  |  | VNI | A |  |
| B57 |  | Clinical |  |  |  |  |  | VNI | A |  |
| B59 |  | Clinical |  |  |  |  |  | VNI | A | ST6 |
| B6 |  | Clinical |  |  |  |  |  | VNI | A |  |
| B61 |  | Clinical |  |  |  |  |  | VNI | A |  |
| B62 |  | Clinical |  |  |  |  |  | VNI | A |  |
| B63 |  | Clinical |  |  |  |  |  | VNI | A |  |
| B64 |  | Clinical |  |  |  |  |  | VNI | A |  |
| B65 |  | Clinical |  |  |  |  |  | VNI | A |  |
| B66 |  | Clinical |  |  |  |  |  | VNI | A |  |
| B67 |  | Clinical |  |  |  |  |  | VNI | A |  |
| B68 |  | Clinical |  |  |  |  |  | VNI | A |  |
| B69 |  | Clinical |  |  |  |  |  | VNI | A |  |
| B70 |  | Clinical |  |  |  |  |  | VNI | A |  |
| B71 |  | Clinical |  |  |  |  |  | VNI | A |  |
| B72 |  | Clinical |  |  |  |  |  | VNI | A |  |
| B73 |  | Clinical |  |  |  |  |  | VNI | A |  |
| B74 |  | Clinical |  |  |  |  |  | VNI | A |  |
| B75 |  | Clinical |  |  |  |  |  | VNI | A |  |
| B76 |  | Clinical |  |  |  |  |  | VNI | A |  |
| B77 |  | Clinical |  |  |  |  |  | VNI | A |  |
| B78 |  | Clinical |  |  |  |  |  | VNI | A |  |
| B79 |  | Clinical |  |  |  |  |  | VNI | A |  |
| B80 |  | Clinical |  |  |  |  |  | VNI | A |  |
| B81 |  | Clinical |  |  |  |  |  | VNI | A |  |
| B82 |  | Clinical |  |  |  |  |  | VNI | A |  |
| B83 |  | Clinical |  |  |  |  |  | VNI | A |  |
| B84 |  | Clinical |  |  |  |  |  | VNI | A |  |
| B85 |  | Clinical |  |  |  |  |  | VNI | A |  |
| B86 |  | Clinical |  |  |  |  |  | VNI | A |  |
| B87 |  | Clinical |  |  |  |  |  | VNI | A |  |
| B88 |  | Clinical |  |  |  |  |  | VNI | A |  |
| B89 |  | Clinical |  |  |  |  |  | VNI | A |  |
| B9 |  | Clinical |  |  |  |  |  | VNI | A |  |
| B90 |  | Clinical |  |  |  |  |  | VNI | A |  |
| B91 |  | Clinical |  |  |  |  |  | VNI | A |  |
| B92 |  | Clinical |  |  |  |  |  | VNI | A |  |
| B95 |  | Clinical |  |  |  |  |  | VNI | A |  |
| C1 | 2004 | Clinical |  |  | M |  |  | VNI | A |  |
| C100 | 2004 | Clinical | CSF | 62 | F |  | Bangkok | VNI | A |  |
| C102 | 2003 | Clinical | CSF | 35 | M | Positive | Srisaket | VNI | A |  |
| C104 | 2003 | Clinical | Blood | 31 | F | Positive | Bangkok | VNI | A |  |
| C105 | 2003 | Clinical |  | 30 | M | Positive | Samutprakarn | VNI | A |  |
| C107 | 2003 | Clinical | CSF | 32 | F | Positive | Bangkok | VNI | A |  |
| C109 | 2003 | Clinical | CSF | 38 | M |  | Khonkaen | VNI | A |  |
| C110 | 2003 | Clinical | CSF | 38 | M |  | Khonkaen | VNI | A | ST4 |
| C114 | 2003 | Clinical |  | 38 | M | Positive | Nonthaburi | VNI | A |  |
| C115 | 2003 | Clinical |  | 32 | F |  | Bangkok | VNI | A |  |
| C116 | 2004 | Clinical | CSF | 36 | M |  | Bangkok | VNI | A |  |
| C117 | 2003 | Clinical | Blood | 31 | F | Positive | Bangkok | VNI | A |  |
| C118 | 2004 | Clinical |  | 28 | F |  | Payao | VNI | A |  |
| C119 | 2003 | Clinical |  | 35 | M | Positive | Srisaket | VNI | A |  |
| C12 | 2004 | Clinical | CSF | 29 | F |  | Bangkok | VNI | A |  |
| C120 | 2003 | Clinical |  | 35 | M | Positive | Srisaket | VNI | A |  |
| C121 | 2003 | Clinical | CSF |  | M |  |  | VNI | A | ST4 |
| C13 | 2004 | Clinical | CSF | 37 | M |  | Samutsongkarm | VNI | A |  |
| C132 | 2003 | Clinical | CSF | 29 | M | Positive |  | VNI | A |  |
| C133 | 2003 | Clinical | CSF |  | M | Positive | Bangkok | VNI | A |  |
| C134 | 2003 | Clinical | CSF | 13 | F | Positive | Bangkok | VNI | A |  |
| C135 | 2003 | Clinical | CSF |  | M |  |  | VNI | A |  |
| C136 | 2003 | Clinical | CSF | 35 | F |  | Nan | VNI | A |  |
| C137 | 2003 | Clinical | CSF | 35 | F |  | Nan | VNI | A |  |
| C138 | 2003 | Clinical | CSF | 21 | M |  | Bangkok | VNI | A |  |
| C139 | 2003 | Clinical | Blood | 21 | M |  | Bangkok | VNI | A |  |
| C14 | 2004 | Clinical | CSF | 37 | M |  | Samutsongkarm | VNI | A |  |
| C142 | 2003 | Clinical | CSF | 51 | M |  | Bangkok | VNI | A |  |
| C143 | 2003 | Clinical |  | 51 | M |  | Bangkok | VNI | A |  |
| C144 | 2003 | Clinical | CSF | 30 | M | Positive | Bangkok | VNI | A |  |
| C145 | 2003 | Clinical | CSF | 30 | M | Positive | Bangkok | VNI | A |  |
| C146 | 2003 | Clinical | CSF | 30 | M | Positive | Bangkok | VNI | A | ST137 |
| C148 | 2003 | Clinical | CSF | 30 | M | Positive | Bangkok | VNI | A |  |
| C149 | 2004 | Clinical | CSF | 52 | M |  | Nakonpathom | VNI | A |  |
| C15 | 2004 | Clinical | CSF | 49 | F |  | Samutprakarn | VNI | A |  |
| C150 | 2003 | Clinical |  | 51 | M |  | Bangkok | VNI | A |  |
| C151 | 2004 | Clinical | CSF | 38 | M |  | Bangkok | VNI | A |  |
| C153 | 2004 | Clinical | CSF | 36 | M |  | Bangkok | VNI | A |  |
| C156 | 2004 | Clinical | CSF |  |  |  |  | VNI | A |  |
| C157 | 2003 | Clinical | CSF | 40 | F | Positive | Bangkok | VNI | A |  |
| C159 | 2003 | Clinical | CSF | 40 | F | Positive | Bangkok | VNI | A |  |
| C16 | 2004 | Clinical | CSF | 31 | F |  | Bangkok | VNI | A |  |
| C161 | 2004 | Clinical | CSF | 61 | M |  | Bangkok | VNI | A |  |
| C164 | 2004 | Clinical | Pus |  | F |  |  | VNI | A |  |
| C165 | 2003 | Clinical | Blood | 34 | M | Positive | Samutprakarn | VNI | A |  |
| C166 | 2004 | Clinical | CSF | 76 | M |  | Bangkok | VNI | A |  |
| C167 | 2003 | Clinical | CSF | 34 | M | Positive | Samutprakarn | VNI | A |  |
| C169 | 2003 | Clinical |  | 34 | M | Positive | Samutprakarn | VNI | A |  |
| C17 | 2004 | Clinical | CSF | 44 | M |  | Nonthaburi | VNI | A |  |
| C171 | 2004 | Clinical | CSF | 38 | M |  | Bangkok | VNI | A |  |
| C173 | 2004 | Clinical | CSF | 30 | M |  |  | VNI | A |  |
| C175 | 2004 | Clinical | CSF |  | M |  |  | VNI | A |  |
| C176 | 2004 | Clinical | CSF | 52 | M |  | Nakonnayok | VNI | A |  |
| C178 | 2004 | Clinical | CSF | 31 | F |  | Chiang Mai | VNI | A |  |
| C179 | 2004 | Clinical |  |  |  |  |  | VNI | A |  |
| C180 | 2003 | Clinical | CSF | 42 | M |  | Bangkok | VNI | A |  |
| C181 | 2003 | Clinical | CSF | 42 | M |  | Bangkok | VNI | A |  |
| C182 | 2003 | Clinical | CSF | 37 | M |  | Bangkok | VNI | A |  |
| C183 | 2003 | Clinical | CSF | 37 | M |  | Bangkok | VNI | A |  |
| C19 | 2004 | Clinical | CSF | 63 | M |  | Bangkok | VNI | A |  |
| C192 | 2004 | Clinical | Blood | 49 | F |  | Nakonpathom | VNI | A |  |
| C194 | 2004 | Clinical | CSF | 60 | M |  | Bangkok | VNI | A |  |
| C195 | 2004 | Clinical | CSF | 45 | M |  | Nakonpanom | VNI | A |  |
| C196 | 2004 | Clinical | CSF | 44 | M |  | Nonthaburi | VNI | A |  |
| C2 | 2004 | Clinical |  |  | F |  |  | VNI | A |  |
| C20 | 2004 | Clinical | Blood | 34 | M |  | Ubolratchatani | VNI | A |  |
| C200 | 2003 | Clinical | CSF | 35 | F |  | Nan | VNI | A |  |
| C201 | 2003 | Clinical | CSF | 42 | M |  | Bangkok | VNI | A |  |
| C203 | 2003 | Clinical | CSF | 30 | M | Positive | Samutprakarn | VNI | A |  |
| C205 | 2004 | Clinical | CSF | 37 | M |  | Bangkok | VNI | A |  |
| C206 | 2004 | Clinical | Blood | 24 | M |  | Bangkok | VNI | A |  |
| C207 | 2004 | Clinical | Blood | 41 | M | Positive | Prachinburi | VNI | A |  |
| C208 | 2004 | Clinical | CSF |  |  |  |  | VNI | A |  |
| C209 | 2003 | Clinical |  | 35 | F |  | Chonburi | VNI | A |  |
| C21 | 2004 | Clinical | CSF | 34 | M |  | Ubolratchatani | VNI | A |  |
| C211 | 2003 | Clinical |  | 42 | M |  | Bangkok | VNI | A |  |
| C22 | 2004 | Clinical | CSF | 34 | M |  | Ubolratchatani | VNI | A |  |
| C23 | 2004 | Clinical | CSF | 34 | M |  | Ubolratchatani | VNI | A |  |
| C24 | 2004 | Clinical | CSF |  | M |  |  | VNI | A |  |
| C25 | 2004 | Clinical |  | 31 | F |  | Bangkok | VNI | A |  |
| C26 | 2004 | Clinical | CSF | 44 | F |  | Bangkok | VNI | A |  |
| C27 | 2004 | Clinical | Blood | 39 | M |  | Bangkok | VNI | A |  |
| C28 | 2004 | Clinical | Blood | 34 | M |  | Ubolratchatani | VNI | A |  |
| C29 | 2004 | Clinical | CSF | 40 | M |  | Suphanburi | VNI | A |  |
| C3 |  | Clinical |  |  | F |  |  | VNI | A |  |
| C30 | 2004 | Clinical | CSF | 44 | F |  | Bangkok | VNI | A |  |
| C31 | 2004 | Clinical | Blood | 31 | F |  | Bangkok | VNI | A |  |
| C32 | 2004 | Clinical | CSF | 31 | F |  | Bangkok | VNI | A |  |
| C33 | 2004 | Clinical | CSF | 31 | F |  | Bangkok | VNI | A |  |
| C34 | 2004 | Clinical | CSF |  | M |  |  | VNI | A |  |
| C35 | 2004 | Clinical | CSF | 29 | M |  | Bangkok | VNI | A |  |
| C36 | 2004 | Clinical | CSF | 29 | F |  | Samutprakarn | VNI | A |  |
| C37 | 2004 | Clinical | Blood | 34 | M |  | Bangkok | VNI | A |  |
| C38 | 2004 | Clinical | CSF | 31 | M |  | Yasothor | VNI | A |  |
| C39 | 2004 | Clinical | Blood | 37 | M |  | Bangkok | VNI | A |  |
| C4 |  | Clinical |  |  | F |  |  | VNI | A |  |
| C40 | 2004 | Clinical | CSF | 34 | M |  | Ubolratchatani | VNI | A |  |
| C41 | 2004 | Clinical | CSF | 34 | M |  | Ubolratchatani | VNI | A |  |
| C42 | 2004 | Clinical | Blood | 45 | M |  | Nakonpanom | VNI | A |  |
| C43 | 2004 | Clinical | CSF | 45 | M |  | Nakonpanom | VNI | A |  |
| C44 | 2004 | Clinical | Blood | 45 | M |  | Nakonpanom | VNI | A |  |
| C45 | 2004 | Clinical | CSF |  |  |  |  | VNI | A |  |
| C46 | 2004 | Clinical | CSF | 45 | M |  | Nakonpanom | VNI | A |  |
| C47 | 2004 | Clinical | CSF | 45 | M |  | Nakonpanom | VNI | A |  |
| C48 | 2004 | Clinical | CSF | 28 | M |  | Bangkok | VNI | A |  |
| C5 |  | Clinical |  |  | F |  |  | VNI | A |  |
| C50 | 2004 | Clinical | CSF |  | M |  |  | VNI | A |  |
| C51 | 2004 | Clinical | CSF |  | M |  |  | VNI | A |  |
| C52 | 2004 | Clinical | Blood | 6 | M |  | Bangkok | VNI | A |  |
| C54 | 2004 | Clinical | CSF | 45 | M |  | Nakonpanom | VNI | A |  |
| C55 | 2004 | Clinical | CSF | 43 | M |  | Chiang Rai | VNI | A |  |
| C56 | 2004 | Clinical | CSF | 43 | M |  | Chiang Rai | VNI | A |  |
| C57 | 2004 | Clinical | CSF | 33 | F |  | Khonkaen | VNI | A |  |
| C58 | 2004 | Clinical |  | 43 | M |  | Chiang Rai | VNI | A |  |
| C59 | 2004 | Clinical | CSF | 33 | F |  | Khonkaen | VNI | A |  |
| C6 | 2004 | Clinical | CSF | 49 | F |  | Samutprakarn | VNI | A |  |
| C60 | 2004 | Clinical |  |  |  |  | Khonkaen | VNI | A |  |
| C61 | 2004 | Clinical | Blood | 39 | M |  | Samutprakarn | VNI | A |  |
| C62 | 2004 | Clinical | CSF | 33 | F |  | Bangkok | VNI | A |  |
| C63 | 2004 | Clinical | CSF | 49 | M |  | Bangkok | VNI | A |  |
| C64 | 2004 | Clinical |  | 33 | F |  | Bangkok | VNI | A |  |
| C67 | 2004 | Clinical | CSF | 34 | M |  | Ubolratchatani | VNI | A |  |
| C68 | 2004 | Clinical | CSF | 38 | M |  | Pichit | VNI | A |  |
| C69 | 2004 | Clinical |  | 38 | M |  | Pichit | VNI | A |  |
| C7 | 2004 | Clinical | CSF | 49 | F |  | Samutprakarn | VNI | A |  |
| C70 | 2004 | Clinical | CSF | 47 | M |  | Bangkok | VNI | A |  |
| C71 | 2004 | Clinical | CSF | 34 | M |  | Ubolratchatani | VNI | A |  |
| C72 | 2004 | Clinical | CSF | 34 | M |  | Ubolratchatani | VNI | A |  |
| C74 | 2004 | Clinical | Blood | 38 | M |  | Nakonnayok | VNI | A |  |
| C75 | 2004 | Clinical | Blood | 26 | F |  | Bangkok | VNI | A |  |
| C76 | 2004 | Clinical | Blood | 26 | F |  | Bangkok | VNI | A |  |
| C77 | 2004 | Clinical | CSF | 38 | M |  | Nakonnayok | VNI | A |  |
| C78 | 2004 | Clinical | CSF | 38 | M |  | Nakonnayok | VNI | A |  |
| C79 | 2003 | Clinical | CSF | 35 | F |  | Chonburi | VNI | A |  |
| C8 | 2004 | Clinical |  | 49 | F |  | Samutprakarn | VNI | A |  |
| C80 | 2004 | Clinical | CSF | 40 | M |  | Umnajcharoen | VNI | A |  |
| C82 | 2004 | Clinical | CSF | 40 | M |  | Umnajcharoen | VNI | A |  |
| C83 | 2003 | Clinical | CSF | 45 | M | Positive |  | VNI | A |  |
| C86 | 2004 | Clinical | CSF | 31 | F |  | Bangkok | VNI | A |  |
| C87 | 2003 | Clinical | CSF |  | M |  |  | VNI | A |  |
| C88 | 2004 | Clinical |  | 33 | M |  | Pathumthani | VNI | A |  |
| C89 | 2004 | Clinical | CSF | 37 | F |  | Pathumthani | VNI | A |  |
| C91 | 2003 | Clinical | CSF | 38 | M | Positive | Nonthaburi | VNI | A |  |
| C93 | 2003 | Clinical |  | 38 | M | Positive | Nonthaburi | VNI | A |  |
| C95 | 2004 | Clinical | Blood | 36 | M |  | Nonthaburi | VNI | A |  |
| C97 | 2003 | Clinical | CSF | 41 | M | Positive | Bangkok | VNI | A |  |
| CM1 |  | Clinical |  |  |  |  |  | VNI | A | ST6 |
| CM14 | 2004 | Clinical |  | 35 | M |  | Chiang Mai | VNI | A |  |
| CM16 | 2004 | Clinical |  | 35 | M |  | Chiang Mai | VNI | A |  |
| CM18 | 2004 | Clinical |  | 33 | M |  | Chiang Mai | VNI | A |  |
| CM19 |  | Environmental |  |  |  |  | Chiang Mai | VNI | A | ST6 |
| CM2 |  | Clinical |  |  |  |  |  | VNI | A |  |
| CM20 |  | Environmental |  |  |  |  | Chiang Mai | VNI | A |  |
| CM3 |  | Clinical |  |  |  |  |  | VNI | A |  |
| CM4 |  | Clinical |  |  |  |  |  | VNI | A |  |
| CM5 |  | Clinical |  |  |  |  |  | VNI | A |  |
| CM6 |  | Clinical |  |  |  |  |  | VNI | A |  |
| CM7 | 2004 | Clinical |  | 36 | F |  | Chiang Mai | VNI | A |  |
| CM8 | 2004 | Clinical |  | 41 | M |  | Chiang Mai | VNI | A |  |
| CM9 | 2004 | Clinical |  | 33 | M |  | Maehongsorn | VNI | A |  |
| E1 | 2003 | Environmental | Bird dropping |  |  |  | Bangkok | VNI | A | ST4 |
| E10 | 2003 | Environmental | Bird dropping |  |  |  | Bangkok | VNI | A |  |
| E11 | 2003 | Environmental | Bird dropping |  |  |  | Bangkok | VNI | A |  |
| E13 | 2003 | Environmental | Bird dropping |  |  |  | Bangkok | VNI | A |  |
| E14 | 2003 | Environmental | Bird dropping |  |  |  | Bangkok | VNI | A |  |
| E15 | 2003 | Environmental | Bird dropping |  |  |  | Bangkok | VNI | A |  |
| E16 | 2003 | Environmental | Bird dropping |  |  |  | Bangkok | VNI | A |  |
| E17 | 2003 | Environmental | Bird dropping |  |  |  | Bangkok | VNI | A |  |
| E18 | 2003 | Environmental | Bird dropping |  |  |  | Bangkok | VNI | A |  |
| E19 | 2003 | Environmental | Bird dropping |  |  |  | Bangkok | VNI | A |  |
| E2 | 2003 | Environmental | Bird dropping |  |  |  | Bangkok | VNI | A |  |
| E20 | 2003 | Environmental | Bird dropping |  |  |  | Bangkok | VNI | A |  |
| E21 | 2003 | Environmental | Bird dropping |  |  |  | Bangkok | VNI | A |  |
| E22 | 2003 | Environmental | Bird dropping |  |  |  | Bangkok | VNI | A |  |
| E23 | 2003 | Environmental | Bird dropping |  |  |  | Bangkok | VNI | A |  |
| E24 | 2003 | Environmental | Bird dropping |  |  |  | Bangkok | VNI | A |  |
| E25 | 2003 | Environmental | Bird dropping |  |  |  | Bangkok | VNI | A |  |
| E26 | 2003 | Environmental | Bird dropping |  |  |  | Bangkok | VNI | A |  |
| E27 | 2003 | Environmental | Bird dropping |  |  |  | Bangkok | VNI | A |  |
| E29 | 2003 | Environmental | Bird dropping |  |  |  | Bangkok | VNI | A |  |
| E3 | 2003 | Environmental | Bird dropping |  |  |  | Bangkok | VNI | A |  |
| E30 | 2003 | Environmental | Bird dropping |  |  |  | Bangkok | VNI | A |  |
| E31 | 2003 | Environmental | Bird dropping |  |  |  | Bangkok | VNI | A |  |
| E32 | 2003 | Environmental | Bird dropping |  |  |  | Bangkok | VNI | A |  |
| E33 | 2003 | Environmental | Bird dropping |  |  |  | Bangkok | VNI | A |  |
| E34 | 2003 | Environmental | Bird dropping |  |  |  | Bangkok | VNI | A |  |
| E35 | 2003 | Environmental | Bird dropping |  |  |  | Bangkok | VNI | A |  |
| E36 | 2003 | Environmental | Bird dropping |  |  |  | Bangkok | VNI | A |  |
| E37 | 2003 | Environmental | Bird dropping |  |  |  | Bangkok | VNI | A |  |
| E39 | 2003 | Environmental | Bird dropping |  |  |  | Bangkok | VNI | A |  |
| E4 | 2003 | Environmental | Bird dropping |  |  |  | Bangkok | VNI | A |  |
| E40 | 2003 | Environmental | Bird dropping |  |  |  | Bangkok | VNI | A |  |
| E41 | 2003 | Environmental | Bird dropping |  |  |  | Bangkok | VNI | A |  |
| E42 | 2003 | Environmental | Bird dropping |  |  |  | Bangkok | VNI | A |  |
| E43 | 2003 | Environmental | Bird dropping |  |  |  | Bangkok | VNI | A |  |
| E44 | 2003 | Environmental | Bird dropping |  |  |  | Bangkok | VNI | A |  |
| E45 | 2003 | Environmental | Bird dropping |  |  |  | Bangkok | VNI | A |  |
| E46 | 2003 | Environmental | Bird dropping |  |  |  | Bangkok | VNI | A |  |
| E47 | 2003 | Environmental | Bird dropping |  |  |  | Bangkok | VNI | A |  |
| E48 | 2003 | Environmental | Bird dropping |  |  |  | Bangkok | VNI | A |  |
| E49 | 2003 | Environmental | Bird dropping |  |  |  | Bangkok | VNI | A |  |
| E5 | 2003 | Environmental | Bird dropping |  |  |  | Bangkok | VNI | A |  |
| E50 | 2003 | Environmental | Bird dropping |  |  |  | Bangkok | VNI | A |  |
| E51 | 2003 | Environmental | Bird dropping |  |  |  | Bangkok | VNI | A |  |
| E52 | 2003 | Environmental | Bird dropping |  |  |  | Bangkok | VNI | A |  |
| E53 | 2003 | Environmental | Bird dropping |  |  |  | Bangkok | VNI | A |  |
| E6 | 2003 | Environmental | Bird dropping |  |  |  | Bangkok | VNI | A |  |
| E7 | 2003 | Environmental | Bird dropping |  |  |  | Bangkok | VNI | A |  |
| E8 | 2003 | Environmental | Bird dropping |  |  |  | Bangkok | VNI | A |  |
| E9 | 2003 | Environmental | Bird dropping |  |  |  | Bangkok | VNI | A |  |
| P10 |  | Clinical |  |  | M |  |  | VNI | A |  |
| P11 |  | Clinical | CSF | 32 | M | Positive |  | VNI | A |  |
| P12 |  | Clinical |  |  |  |  |  | VNI | A |  |
| P13 |  | Clinical | CSF | 27 | M | Positive |  | VNI | A |  |
| P14 |  | Clinical |  |  |  |  |  | VNI | A |  |
| P15 |  | Clinical | Blood | 56 | F |  |  | VNI | A |  |
| P16 |  | Clinical | CSF | 27 | M | Positive |  | VNI | A |  |
| P17 |  | Clinical | CSF | 35 | M | Positive |  | VNI | A | ST4 |
| P18 |  | Clinical |  |  |  |  |  | VNI | A |  |
| P19 |  | Clinical | Blood | 36 | F | Positive |  | VNI | A |  |
| P2 |  | Clinical | CSF | 36 | M | Positive |  | VNI | A |  |
| P20 |  | Clinical | CSF | 35 | M | Positive |  | VNI | A |  |
| P4 |  | Clinical |  |  |  |  |  | VNI | A | ST4 |
| P6 |  | Clinical |  |  | F |  |  | VNI | A |  |
| P7 |  | Clinical |  |  |  |  |  | VNI | A |  |
| P8 |  | Clinical |  |  | F |  |  | VNI | A |  |
| P9 |  | Clinical |  |  |  |  |  | VNI | A |  |
| S1 |  | Clinical |  |  |  |  |  | VNI | A |  |
| S10 |  | Clinical |  |  |  |  |  | VNI | A |  |
| S11 |  | Clinical |  |  |  |  |  | VNI | A |  |
| S12 |  | Clinical |  |  |  |  |  | VNI | A |  |
| S13 |  | Clinical |  |  |  |  |  | VNI | A |  |
| S14 |  | Clinical |  |  |  |  |  | VNI | A |  |
| S15 |  | Clinical |  |  |  |  |  | VNI | A |  |
| S16 |  | Clinical |  |  |  |  |  | VNI | A |  |
| S18 |  | Clinical |  |  |  |  |  | VNI | A |  |
| S19 |  | Clinical |  |  |  |  |  | VNI | A |  |
| S2 |  | Clinical |  |  |  |  |  | VNI | A |  |
| S20 |  | Clinical |  |  |  |  |  | VNI | A |  |
| S21 |  | Clinical |  |  |  |  |  | VNI | A |  |
| S23 |  | Clinical |  |  |  |  |  | VNI | A |  |
| S24 |  | Clinical |  |  |  |  |  | VNI | A | ST6 |
| S25 |  | Clinical |  |  |  |  |  | VNI | A |  |
| S26 |  | Clinical |  |  |  |  |  | VNI | A |  |
| S27 |  | Clinical |  |  |  |  |  | VNI | A |  |
| S28 |  | Clinical |  |  |  |  |  | VNI | A |  |
| S29 |  | Clinical |  |  |  |  |  | VNI | A |  |
| S30 |  | Clinical |  |  |  |  |  | VNI | A |  |
| S31 |  | Clinical |  |  |  |  |  | VNI | A |  |
| S33 |  | Clinical |  |  |  |  |  | VNI | A |  |
| S34 |  | Clinical |  |  |  |  |  | VNI | A |  |
| S35 |  | Clinical |  |  |  |  |  | VNI | A |  |
| S36 |  | Clinical |  |  |  |  |  | VNI | A |  |
| S37 |  | Clinical |  |  |  |  |  | VNI | A |  |
| S38 |  | Clinical |  |  |  |  |  | VNI | A |  |
| S4 |  | Clinical |  |  |  |  |  | VNI | A |  |
| S40 |  | Clinical |  |  |  |  |  | VNI | A |  |
| S42 |  | Clinical |  |  |  |  |  | VNI | A |  |
| S43 |  | Clinical |  |  |  |  |  | VNI | A |  |
| S44 |  | Clinical |  |  |  |  |  | VNI | A |  |
| S45 |  | Clinical |  |  |  |  |  | VNI | A |  |
| S46 |  | Clinical |  |  |  |  |  | VNI | A |  |
| S47 |  | Clinical |  |  |  |  |  | VNI | A |  |
| S48 |  | Clinical |  |  |  |  |  | VNI | A |  |
| S49 |  | Clinical |  |  |  |  |  | VNI | A |  |
| S5 |  | Clinical |  |  |  |  |  | VNI | A |  |
| S50 |  | Clinical |  |  |  |  |  | VNI | A |  |
| S51 |  | Clinical |  |  |  |  |  | VNI | A |  |
| S54 |  | Clinical |  |  |  |  |  | VNI | A |  |
| S55 |  | Clinical |  |  |  |  |  | VNI | A |  |
| S56 |  | Clinical |  |  |  |  |  | VNI | A |  |
| S57 |  | Clinical |  |  |  |  |  | VNI | A |  |
| S59 |  | Clinical |  |  |  |  |  | VNI | A |  |
| S6 |  | Clinical |  |  |  |  |  | VNI | A |  |
| S60 |  | Clinical |  |  |  |  |  | VNI | A |  |
| S61 |  | Clinical |  |  |  |  |  | VNI | A |  |
| S62 |  | Clinical |  |  |  |  |  | VNI | A |  |
| S63 |  | Clinical |  |  |  |  |  | VNI | A |  |
| S64 |  | Clinical |  |  |  |  |  | VNI | A |  |
| S65 |  | Clinical |  |  |  |  |  | VNI | A |  |
| S66 |  | Clinical |  |  |  |  |  | VNI | A |  |
| S68 |  | Clinical |  |  |  |  |  | VNI | A |  |
| S69 |  | Clinical |  |  |  |  |  | VNI | A |  |
| S7 |  | Clinical |  |  |  |  |  | VNI | A |  |
| S70 |  | Clinical |  |  |  |  |  | VNI | A |  |
| S71 |  | Clinical |  |  |  |  |  | VNI | A |  |
| S72 |  | Clinical |  |  |  |  |  | VNI | A |  |
| S73 |  | Clinical |  |  |  |  |  | VNI | A |  |
| S75 |  | Clinical |  |  |  |  |  | VNI | A |  |
| S77 |  | Clinical |  |  |  |  |  | VNI | A |  |
| S78 |  | Clinical |  |  |  |  |  | VNI | A |  |
| S79 |  | Clinical |  |  |  |  |  | VNI | A |  |
| S8 |  | Clinical |  |  |  |  |  | VNI | A |  |
| S80 |  | Clinical |  |  |  |  |  | VNI | A |  |
| S81 |  | Clinical |  |  |  |  |  | VNI | A |  |
| S82 |  | Clinical |  |  |  |  |  | VNI | A |  |
| S83 |  | Clinical |  |  |  |  |  | VNI | A |  |
| S84 |  | Clinical |  |  |  |  |  | VNI | A |  |
| S85 |  | Clinical |  |  |  |  |  | VNI | A |  |
| S88 |  | Clinical |  |  |  |  |  | VNI | A |  |
| S89 |  | Clinical |  |  |  |  |  | VNI | A |  |
| S9 |  | Clinical |  |  |  |  |  | VNI | A |  |
| S90 |  | Clinical |  |  |  |  |  | VNI | A |  |
| S91 |  | Clinical |  |  |  |  |  | VNI | A |  |
| S92 |  | Clinical |  |  |  |  |  | VNI | A |  |
| S93 |  | Clinical |  |  |  |  |  | VNI | A |  |
| S94 |  | Clinical |  |  |  |  |  | VNI | A |  |
| S95 |  | Clinical |  |  |  |  |  | VNI | A |  |
| T1 | 2005 | Environmental | Bird dropping |  |  |  | Bangkok | VNI | A |  |
| T11 | 2005 | Environmental | Bird dropping |  |  |  | Bangkok | VNI | A |  |
| T12 | 2005 | Environmental | Bird dropping |  |  |  | Bangkok | VNI | A |  |
| T13 | 2005 | Environmental | Bird dropping |  |  |  | Bangkok | VNI | A |  |
| T14 | 2005 | Environmental | Bird dropping |  |  |  | Bangkok | VNI | A |  |
| T15 | 2005 | Environmental | Bird dropping |  |  |  | Bangkok | VNI | A |  |
| T17 | 2005 | Environmental | Bird dropping |  |  |  | Bangkok | VNI | A |  |
| T18 | 2005 | Environmental | Bird dropping |  |  |  | Bangkok | VNI | A |  |
| T19 | 2005 | Environmental | Bird dropping |  |  |  | Bangkok | VNI | A |  |
| T2 | 2005 | Environmental | Bird dropping |  |  |  | Bangkok | VNI | A |  |
| T20 | 2005 | Environmental | Bird dropping |  |  |  | Bangkok | VNI | A |  |
| T21 | 2005 | Environmental | Bird dropping |  |  |  | Bangkok | VNI | A |  |
| T22 | 2005 | Environmental | Bird dropping |  |  |  | Bangkok | VNI | A |  |
| T23 | 2005 | Environmental | Bird dropping |  |  |  | Bangkok | VNI | A |  |
| T24 | 2005 | Environmental | Bird dropping |  |  |  | Bangkok | VNI | A |  |
| T25 | 2005 | Environmental | Bird dropping |  |  |  | Bangkok | VNI | A |  |
| T26 | 2005 | Environmental | Bird dropping |  |  |  | Bangkok | VNI | A |  |
| T27 | 2005 | Environmental | Bird dropping |  |  |  | Bangkok | VNI | A |  |
| T28 | 2005 | Environmental | Bird dropping |  |  |  | Bangkok | VNI | A | ST6 |
| T3 | 2005 | Environmental | Bird dropping |  |  |  | Bangkok | VNI | A |  |
| T30 | 2005 | Environmental | Bird dropping |  |  |  | Bangkok | VNI | A |  |
| T31 | 2005 | Environmental | Bird dropping |  |  |  | Bangkok | VNI | A |  |
| T32 | 2005 | Environmental | Bird dropping |  |  |  | Bangkok | VNI | A |  |
| T4 | 2005 | Environmental | Bird dropping |  |  |  | Bangkok | VNI | A |  |
| T5 | 2005 | Environmental | Bird dropping |  |  |  | Bangkok | VNI | A |  |
| T6 | 2005 | Environmental | Bird dropping |  |  |  | Bangkok | VNI | A |  |
| T8 | 2005 | Environmental | Bird dropping |  |  |  | Bangkok | VNI | A |  |
| T9 | 2005 | Environmental | Bird dropping |  |  |  | Bangkok | VNI | A |  |
| A19 | 2005 | Veterinary | Eye |  |  |  |  | VNI | B | ST31 |
| ST31 | 2005 | Veterinary | Eye |  |  |  |  | VNI | C |  |
| A18 | 2005 | Veterinary | Eye |  |  |  |  | VNI | C | ST31 |
| A20 | 2005 | Veterinary | Eye |  |  |  |  | VNI | C |  |
| C101 | 2003 | Clinical | CSF | 34 | M | Positive | Bangkok | VNI | D |  |
| C106 | 2003 | Clinical |  | 34 | M | Positive | Bangkok | VNI | D |  |
| C108 | 2003 | Clinical | Blood | 13 | F | Positive | Bangkok | VNI | D |  |
| C111 | 2003 | Clinical | CSF | 13 | F | Positive | Bangkok | VNI | D |  |
| C113 | 2003 | Clinical |  | 13 | F | Positive | Bangkok | VNI | D |  |
| E12 | 2003 | Environmental | Bird dropping |  |  |  | Bangkok | VNI | D | ST77 |
| S76 |  | Clinical |  |  |  |  |  | VNI | D |  |
| S86 |  | Clinical |  |  |  |  |  | VNI | D |  |
| S87 |  | Clinical |  |  |  |  |  | VNI | D | ST3 |
| B31 |  | Clinical |  |  |  |  |  | VNI | E | ST4 |
| A1 | 2005 | Veterinary | Nose |  |  |  |  | VNI | F |  |
| A2 | 2005 | Veterinary | Nose |  |  |  |  | VNI | F |  |
| A29 | 2005 | Veterinary | blood |  |  |  |  | VNI | F |  |
| A3 | 2005 | Veterinary | Nose |  |  |  |  | VNI | F |  |
| A30 | 2005 | Veterinary | lung |  |  |  |  | VNI | F |  |
| A31 | 2005 | Veterinary | kidney |  |  |  |  | VNI | F |  |
| A32 | 2005 | Veterinary | blood |  |  |  |  | VNI | F |  |
| A4 | 2005 | Veterinary | Nose |  |  |  |  | VNI | F |  |
| B12 |  | Clinical |  |  |  |  |  | VNI | F |  |
| B2 |  | Clinical |  |  |  |  |  | VNI | F | ST5 |
| B3 |  | Clinical |  |  |  |  |  | VNI | F |  |
| B42 |  | Clinical |  |  |  |  |  | VNI | F |  |
| B48 |  | Clinical |  |  |  |  |  | VNI | F |  |
| B5 |  | Clinical |  |  |  |  |  | VNI | F |  |
| B58 |  | Clinical |  |  |  |  |  | VNI | F |  |
| C10 | 2004 | Clinical | CSF | 36 | F |  | Samutprakarn | VNI | F |  |
| C11 | 2004 | Clinical | CSF | 40 | M |  | Khalasin | VNI | F |  |
| C140 | 2003 | Clinical | CSF |  | M |  |  | VNI | F | ST81 |
| C154 | 2004 | Clinical | CSF |  | M |  |  | VNI | F |  |
| C65 | 2004 | Clinical |  | 47 | M |  | Bangkok | VNI | F |  |
| C66 | 2004 | Clinical |  | 47 | M |  | Bangkok | VNI | F |  |
| C73 | 2004 | Clinical | Sinus |  |  |  |  | VNI | F |  |
| C81 | 2004 | Clinical |  | 36 | F |  | Bangkok | VNI | F |  |
| CM10 | 2004 | Clinical |  | 30 | M |  | Lopburi | VNI | F |  |
| CM11 | 2004 | Clinical |  | 59 | M |  | Payao | VNI | F |  |
| CM12 | 2004 | Clinical |  | 30 | M |  | Lopburi | VNI | F |  |
| CM13 | 2004 | Clinical |  | 59 | M |  | Payao | VNI | F |  |
| CM15 | 2004 | Clinical |  | 30 | M |  | Lopburi | VNI | F |  |
| CM17 | 2004 | Clinical |  | 32 | F |  | Chiang Mai | VNI | F | ST5 |
| E38 | 2003 | Environmental | Bird dropping |  |  |  | Bangkok | VNI | F | ST5 |
| P21 |  | Clinical | CSF |  | F | Positive |  | VNI | F |  |
| P3 |  | Clinical | Blood | 31 | F |  |  | VNI | F |  |
| 46-2852 | 2003 | Clinical | CSF | 39 | M |  |  | VNII |  | ST42 |
| 47-1104 | 2004 | Clinical | CSF | 39 | F | Positive | Bangkok | VNII |  | ST40 |
| 47-7559 | 2004 | Clinical | Blood | 69 | F |  |  | VNII |  | ST40 |
| 48-1350 | 2005 | Clinical | Blood | 36 | M | Positive | Bangkok | VNII |  | ST40 |
| 48-1398 | 2005 | Clinical | CSF | 36 | M | Positive | Bangkok | VNII |  | ST40 |
| 48-1643 | 2005 | Clinical | CSF |  | M |  |  | VNII |  | ST40 |
| 48-1663 | 2005 | Clinical | CSF | 36 | M | Positive | Bangkok | VNII |  | ST40 |
| 48-2323 | 2005 | Clinical | BAL | 60 | M | Negative | Bangkok | VNII |  | ST43 |
| A10 | 2005 | Veterinary | Nose |  |  |  |  | VNII |  | ST43 |
| A11 | 2005 | Veterinary | Nose |  |  |  |  | VNII |  | ST43 |
| A5 | 2005 | Veterinary | Nose |  |  |  |  | VNII |  | ST172 |
| A6 | 2005 | Veterinary | Nose |  |  |  |  | VNII |  | ST172 |
| CBS7816 |  | Environmental | Bird dropping |  |  |  | Chantaburi | VNIV |  | ST126 |

*These samples are anonymous and the data cannot be used to trace back to individuals.
