# Supplementary material for: Molecular Epidemiology Reveals Genetic Diversity amongst Isolates of the Cryptococcus neoformans/C. gattii Species Complex in Thailand
Source: PLoS Negl Trop Dis. 2013 Jul 4;7(7):e2297. doi: 10.1371/journal.pntd.0002297 (PMC3701708; doi:10.1371/journal.pntd.0002297)
Supplement: Table S2 — Correlation between old and new allele and sequence type numbering from the Simwami et al. 2011 (28) publication and the new C. gattii MLST database at mlst.mycologylab.com for the MLST data used in the current study. (DOC) [file pntd.0002297.s003.doc]

**Table S2:** Correlation between old and new allele and sequence type numbering from the Simwami *et al.* 2011 (33) publication and the new *C. gattii* MLST database at mlst.mycologylab.com for the MLST data used in the current study

| **Strain Number** | ***CAP59*** | | ***GPD1*** | | **IGS1** | | ***LAC1*** | | ***PLB1*** | | ***SOD1*** | | ***URA5*** | | **ST** | |
| --- | --- | --- | --- | --- | --- | --- | --- | --- | --- | --- | --- | --- | --- | --- | --- | --- |
|  | **Old** | **New** | **Old** | **New** | **Old** | **New** | **Old** | **New** | **Old** | **New** | **Old** | **New** | **Old** | **New** | **Old** | **New** |
| CN5019 | 1 | **1** | 1 | **1** | 19 | **1** | 4 | **4** | 2 | **2** | 13 | **1** | 5 | **5** | 45 | **4** |
| CN49004 | 1 | **1** | 3 | **3** | 19 | **1** | 5 | **5** | 2 | **2** | 13 | **1** | 1 | **1** | 46 | **5** |
| CN5010 | 1 | **1** | 1 | **1** | 19 | **1** | 3 | **3** | 2 | **2** | 13 | **1** | 5 | **5** | 44 | **6** |
| CN5007 | 1 | **1** | 1 | **23** | 20 | **10** | 3 | **3** | 4 | **4** | 13 | **1** | 1 | **1** | 47 | **93** |
| Pt 5 | 1 | **1** | 1 | **1** | 19 | **1** | 5 | **5** | 2 | **2** | 13 | **1** | 1 | **1** | 51 | **81** |
| 4_9 | 1 | **1** | 1 | **1** | 19 | **1** | 9 | **9** | 2 | **2** | 13 | **1** | 5 | **5** | 52 | **82** |
| K 45 | 1 | **1** | 1 | **1** | 19 | **1** | 3 | **3** | 4 | **4** | 13 | **1** | 5 | **5** | 50 | **83** |
| D 9 | 1 | **1** | 1 | **1** | 19 | **1** | 4 | **4** | 2 | **2** | 13 | **1** | 14 | **14** | 49 | **141** |
| CM 21 | 2 | **2** | 10 | **10** | 14 | **14** | 6 | **6** | 11 | **11** | 14 | **11** | 4 | **4** | 48 | **173** |
