# Supplementary material for: Molecular Epidemiology Reveals Genetic Diversity amongst Isolates of the Cryptococcus neoformans/C. gattii Species Complex in Thailand
Source: PLoS Negl Trop Dis. 2013 Jul 4;7(7):e2297. doi: 10.1371/journal.pntd.0002297 (PMC3701708; doi:10.1371/journal.pntd.0002297)
Supplement: Table S3 — MLST data for the additional published C. neoformans strains used in this study. (DOC) [file pntd.0002297.s004.doc]

**Table S3**. MLST data for the additional published *C. neoformans* strains used in this study

| **Molecular type** | **Strain** | **Country** | ***CAP59*** | ***GPD1*** | **IGS1** | ***LAC1*** | ***PLB1*** | ***SOD1*** | ***URA5*** | **ST** | **Reference** |
| --- | --- | --- | --- | --- | --- | --- | --- | --- | --- | --- | --- |
| VNB | bt31 | Africa | 4 | 11 | 9 | 6 | 7 | 1 | 12 | 8 | (22) |
|  | bt109 | Africa | 4 | 11 | 7 | 6 | 4 | 1 | 3 | 9 | (22) |
|  | bt65 | Africa | 9 | 11 | 2 | 6 | 4 | 1 | 3 | 10 | (22) |
|  | bt89 | Africa | 4 | 11 | 2 | 6 | 4 | 7 | 3 | 16 | (22) |
|  | bt84 | Africa | 5 | 11 | 16 | 6 | 6 | 5 | 3 | 17 | (22) |
| VNI | H99 | USA | 7 | 1 | 1 | 1 | 1 | 1 | 2 | 2 | (23) |
|  | CN5019 | Thailand | 1 | 1 | 1 | 4 | 2 | 1 | 5 | 4 | (33) |
|  | CN49004 | Thailand | 1 | 3 | 1 | 5 | 2 | 1 | 1 | 5 | (33) |
|  | CN5010 | Thailand | 1 | 1 | 1 | 3 | 2 | 1 | 5 | 6 | (33) |
|  | A2-102-5 | USA | 1 | 2 | 6 | 3 | 2 | 1 | 1 | 15 | (22) |
|  | ug2467 | Africa | 7 | 1 | 1 | 2 | 1 | 1 | 2 | 23 | (22) |
|  | ug2471 | Africa | 1 | 1 | 10 | 3 | 2 | 1 | 1 | 31 | (22) |
|  | A4-34-6 | USA | 1 | 1 | 11 | 3 | 4 | 1 | 1 | 39 | (22) |
|  | A4-1-12 | USA | 1 | 1 | 1 | 18 | 1 | 1 | 2 | 58 | (22) |
|  | A3-38-20 | USA | 7 | 1 | 1 | 18 | 1 | 1 | 1 | 63 | (22) |
|  | bt100 | Africa | 7 | 5 | 1 | 18 | 5 | 2 | 9 | 73 | (22) |
|  | bt134 | Africa | 1 | 3 | 46 | 5 | 2 | 1 | 1 | 79 | (22) |
|  | Pt 5 | Thailand | 1 | 1 | 1 | 5 | 2 | 1 | 1 | 81 | (33) |
|  | 4_9 | Thailand | 1 | 1 | 1 | 9 | 2 | 1 | 5 | 82 | (33) |
|  | K 45 | Thailand | 1 | 1 | 1 | 3 | 4 | 1 | 5 | 83 | (33) |
|  | bt68 | Africa | 1 | 5 | 1 | 18 | 4 | 1 | 1 | 87 | (22) |
|  | CN5007 | Thailand | 1 | 23 | 10 | 3 | 4 | 1 | 1 | 93 | (33) |
|  | c26 | USA | 7 | 1 | 1 | 2 | 1 | 3 | 2 | 103 | (22) |
|  | D 9 | Thailand | 1 | 1 | 1 | 4 | 2 | 1 | 14 | 141 | (33) |
| VNII | c45 | USA | 10 | 9 | 14 | 8 | 11 | 12 | 4 | 41 | (22) |
|  | c44 | USA | 8 | 10 | 15 | 8 | 12 | 3 | 11 | 42 | (22) |
|  | A7-35-23 | USA | 2 | 9 | 14 | 8 | 11 | 11 | 4 | 43 | (23) |
|  | c16 | USA | 2 | 9 | 14 | 8 | 16 | 11 | 4 | 60 | (22) |
|  | ug2472 | Africa | 2 | 9 | 51 | 8 | 11 | 12 | 4 | 100 | (22) |
|  | CM 21 | Thailand | 2 | 10 | 14 | 6 | 11 | 11 | 4 | 173 | (33) |
| VNI Standard | WM 148 | Australia | 7 | 1 | 1 | 18 | 1 | 1 | 1 | 63 | (35) |
| VNII Standard | WM 626 | Australia | 2 | 14 | 14 | 8 | 11 | 11 | 27 | 97 | (35) |
| VNIV Standard | WM 629 | Australia | 16 | 21 | 30 | 19 | 13 | 1 | 19 | 117 | (35) |
