# Supplementary material for: Molecular Epidemiology Reveals Genetic Diversity amongst Isolates of the Cryptococcus neoformans/C. gattii Species Complex in Thailand
Source: PLoS Negl Trop Dis. 2013 Jul 4;7(7):e2297. doi: 10.1371/journal.pntd.0002297 (PMC3701708; doi:10.1371/journal.pntd.0002297)
Supplement: Table S4 — GenBank accession numbers for all sequences of the MLST alleles obtained from Thai C. neoformans and C. gattii isolates used in this study. (DOC) [file pntd.0002297.s005.doc]

**Table S4.** GenBank accession numbers for all sequences of the MLST alleles obtained from Thai *C. neoformans* and *C. gattii* isolates used in this study

| **Species** | **Loci** | **Alleles** | **GenBank Accession number** |
| --- | --- | --- | --- |
| *Cryptococcus neoformans* | ***CAP59*** | *CAP59* allele 1 | KC292381 |
|  |  | *CAP59* allele 2 | KC292382 |
|  |  | *CAP59* allele 8 | KC292383 |
|  |  | *CAP59* allele 17 | KC292384 |
|  | ***GPD1*** | *GPD1* allele 1 | KC292385 |
|  |  | *GPD1* allele 3 | KC292386 |
|  |  | *GPD1* allele 9 | KC292387 |
|  |  | *GPD1* allele 10 | KC292388 |
|  |  | *GPD1* allele 21 | KC292389 |
|  | **IGS1** | IGS1 allele 1 | KC292415 |
|  |  | IGS1 allele 10 | KC292416 |
|  |  | IGS1 allele 14 | KC292417 |
|  |  | IGS1 allele 15 | KC292418 |
|  |  | IGS1 allele 25 | KC292419 |
|  |  | IGS1 allele 28 | KC292420 |
|  | ***LAC1*** | *LAC1* allele 3 | KC292390 |
|  |  | *LAC1* allele 4 | KC292391 |
|  |  | *LAC1* allele 5 | KC292392 |
|  |  | *LAC1* allele 6 | KC292393 |
|  |  | *LAC1* allele 8 | KC292394 |
|  |  | *LAC1* allele 9 | KC292395 |
|  |  | *LAC1* allele 11 | KC292396 |
|  |  | *LAC1* allele 19 | KC292397 |
|  | ***PLB1*** | *PLB1* allele 2 | KC292398 |
|  |  | *PLB1* allele 4 | KC292399 |
|  |  | *PLB1* allele 11 | KC292400 |
|  |  | *PLB1* allele 12 | KC292401 |
|  |  | *PLB1* allele 14 | KC292402 |
|  | ***SOD1*** | *SOD1* allele 1 | KC292403 |
|  |  | *SOD1* allele 3 | KC292404 |
|  |  | *SOD1* allele 11 | KC292405 |
|  |  | *SOD1* allele 12 | KC292406 |
|  |  | *SOD1* allele 16 | KC292407 |
|  | ***URA5*** | *URA5* allele 1 | KC292408 |
|  |  | *URA5* allele 4 | KC292409 |
|  |  | *URA5* allele 5 | KC292410 |
|  |  | *URA5* allele 11 | KC292411 |
|  |  | *URA5* allele 14 | KC292412 |
|  |  | *URA5* allele 15 | KC292413 |
|  |  | *URA5* allele 20 | KC292414 |
| *Cryptococcus gattii* | ***CAP59*** | *CAP59* allele 2 | GU079817 |
|  | ***GPD1*** | *GPD1* allele 6 | GU079849 |
|  | **IGS1** | IGS1 allele 10 | GU079880 |
|  |  | IGS1 allele 32 | KC306863 |
|  | ***LAC1*** | *LAC1* allele 4 | GU079911 |
|  | ***PLB1*** | *PLB1* allele 2 | GU079699 |
|  | ***SOD1*** | *SOD1* allele 15 | GU079730 |
|  | ***URA5*** | *URA5* allele 2 | GU079773 |
